# A DUF4148 family protein produced inside RAW264.7 cells is a critical Burkholderia pseudomallei virulence factor

**DOI:** 10.1080/21505594.2020.1806675

**Published:** 2020-08-23

**Authors:** Susan Welkos, Irma Blanco, Udoka Okaro, Jennifer Chua, David DeShazer

**Affiliations:** Bacteriology Division, United States Army Medical Research Institute of Infectious Diseases, Frederick, Maryland, USA

**Keywords:** Domain of unknown function, facultative intracellular pathogen, select agent

## Abstract

Burkholderia pseudomallei: is the etiological agent of the disease melioidosis and is a Tier 1 select agent. It survives and replicates inside phagocytic cells by escaping from the endocytic vacuole, replicating in the cytosol, spreading to other cells via actin polymerization and promoting the fusion of infected and uninfected host cells to form multinucleated giant cells. In this study, we utilized a proteomics approach to identify bacterial proteins produced inside RAW264.7 murine macrophages and host proteins produced in response to *B. pseudomallei* infection. Cells infected with *B. pseudomallei* strain K96243 were lysed and the lysate proteins digested and analyzed using nanoflow reversed-phase liquid chromatography and tandem mass spectrometry. Approximately 160 bacterial proteins were identified in the infected macrophages, including BimA, TssA, TssB, Hcp1 and TssM. Several previously uncharacterized *B. pseudomallei* proteins were also identified, including BPSS1996 and BPSL2748. Mutations were constructed in the genes encoding these novel proteins and their relative virulence was assessed in BALB/c mice. The 50% lethal dose for the *BPSS1996* mutant was approximately 55-fold higher than that of the wild type, suggesting that BPSS1996 is required for full virulence. Sera from *B. pseudomallei*-infected animals reacted with BPSS1996 and it was found to localize to the bacterial surface using indirect immunofluorescence. Finally, we identified 274 host proteins that were exclusively present or absent in infected RAW264.7 cells, including chemokines and cytokines involved in controlling the initial stages of infection.

## Introduction

Melioidosis, an infection of humans and animals, is caused by the Gram-negative bacterium *Burkholderia pseudomallei* [1,2]. The disease occurs throughout the tropics, but is especially prevalent in Southeast Asia and northern Australia. The organism can be isolated from soil and water in endemic regions and infections occur following contact with environmental sources via inhalation, ingestion or cutaneous inoculation. Infections can be latent, chronic or acute and disease manifestation is largely dependent on the immune status of the host, the route of infection and the infectious dose. *B. pseudomallei* is designated as a Tier 1 Select Agent in the United States because of its potential for misuse as a biological weapon [3]. No licensed melioidosis vaccine is currently available and antibiotic treatment can be challenging because the organism is naturally resistant to many antimicrobial agents [4,5].

*B. pseudomallei* possesses a relatively large genome (~7 Mb) consisting of two chromosomes and encoding nearly 6,000 proteins [6,7]. Numerous virulence determinants have been identified, but relatively little is known about the molecular pathogenesis of *B. pseudomallei* infection [8]. The microbe is a facultative intracellular pathogen that can survive and replicate inside of host phagocytic cells [2]. It employs multiple virulence factors that interfere with a macrophage’s innate ability to efficiently eliminate infection. The goal of this study was to identify bacterial proteins produced inside RAW264.7 murine macrophages and examine host cell proteins that are increased or decreased in response to *B. pseudomallei* infection.

## Materials and methods

### Bacterial strains, plasmids, and growth conditions

*Escherichia coli* and *B. pseudomallei* K96243 [6] and MSHR668 [9] were grown at 37°C on LB agar (Lennox formulation) or in Luria-Bertani broth (LB) (Lennox formulation), with 100 μg/ml adenine HCl and 5 μg/ml thiamine HCl for the *purM* select agent exempt strain Bp82 [10]. When appropriate, antibiotics were added at the following concentrations: 25 µg/ml kanamycin (Km) and streptomycin (Sm) for *E. coli* and 25 µg/ml polymyxin B (Pm) and 500–1000 µg/ml Km for *B. pseudomallei*. For induction studies, isopropyl-ß-D-1-thiogalactopyranoside (IPTG) was added to a final concentration of 0.5 mM. A 20 mg/ml stock solution of the chromogenic indicator 5-bromo-4-chloro-3-indolyl-ß-D-galactopyranoside (X-Gal) was prepared in *N,N*-dimethylformamide, and 40 μl was spread onto the surface of plate medium for blue/white screening in *E. coli* TOP10 or *E. cloni*® 10 G chemically competent cells. All manipulations with *B. pseudomallei* select agent strains were carried out in a class II microbiological safety cabinet located in a designated biosafety level 3 (BSL-3) laboratory. Other strains were handled in in a class II microbiological safety cabinet located in a designated BSL-2 laboratory.

### Sample preparation prior to mass spectrometric analysis

Proteins from control and infected cell lysates were extracted and quantitated using a BCA assay (ThermoFisher Scientific, Cat. No. 23225). Equal amounts of protein from samples prepared on two separate occasions were reduced, alkylated and trypsin digested overnight using an enzyme-to-protein ratio of 1:50. Tryptic peptides were further desalted using a C18 spin column. One twentieth of each sample was lyophilized and reconstituted in 0.1% trifluoroacetic acid (TFA) and analyzed in quadruplicate without fractionation for quantitation. The remainder of the tryptic peptides were lyophilized dissolved in 25% acetonitrile with 0.1% formic acid and further fractionated using strong cation-exchange (SCX) chromatography [11]. The SCX fractions of the two samples were pooled into 10 fractions each, lyophilized and reconstituted in 0.1% TFA to be analyzed by liquid chromatography mass spectrometry (LCMS).

### Nanobore reversed-phase liquid chromatography tandem MS (nanoRPLC-MS/MS)

NanoRPLC-MS/MS was performed using an Agilent 1200 nanoflow LC system coupled online with a LTQ Orbitrap Velos mass spectrometer. The RPLC column (75 µm i.d. x 10 cm) were slurry-packed in-house with 5 µm, 300 Å pore size C-18 stationary phase into fused silica capillaries with a flame pulled tip. After sample injection, the column was washed for 20 min with 98% mobile phase A (0.1% formic acid in water) at 0.5 µl min^−1^. Peptides were eluted using a linear gradient of 2% mobile phase B (0.1% formic acid in ACN) to 35% B in 100 minutes, then to 80% B over an additional 20 minutes. The column flow-rate was maintained at 0.25 µl min^−1^ throughout the separation gradient. The mass spectrometer was operated in a data-dependent mode in which each full MS scan was followed by ten MS/MS scans wherein the ten most abundant molecular ions were dynamically selected for collision-induced dissociation (CID) using a normalized collision energy of 35%.

### Identification and quantification of proteins from nanoRPLC-MS/MS

The RPLC-MS/MS data were processed using MaxQuant software (version 1.2.2.5). MS/MS data were searched by the Andromeda search engine against a combined decoy database of mouse database and *B. pseudomallei* database containing both forward and reverse sequences. Dynamic modifications of methionine oxidation and N-terminal acetylation as well as fixed modification of carbamidomethyl cysteine were also included in the database search. Only tryptic peptides with up to two missed cleavage sites with a minimum peptide length of six amino acids were allowed. The false discovery rate (FDR) was set to 0.01 for both peptide and protein identifications. Mouse protein changes due to *B. pseudomallei* infection were analyzed using label-free quantification (LFQ) function of MaxQuant. Quantitation results were further analyzed using Perseus program (version 1.2.0.17). Significant protein changes were determined using a two sample t-test, protein intensity replicates for the two samples were grouped and the statistical test was performed with an FDR value of 0.001.

### DNA manipulation

Restriction enzymes (Roche Molecular Biochemicals), Antarctic phosphatase (New England BioLabs, Cat. No. M0289S), and T4 DNA ligase (Roche Molecular Biochemicals, Cat. No. 10481220001) were used according to the manufacturer’s instructions. When necessary, the End-It DNA End-Repair Kit (Lucigen, Cat. No. ER0720) was used to convert 5ʹ or 3ʹ protruding ends to blunt-ended DNA. DNA fragments used in cloning procedures were excised from agarose gels and purified with a GENECLEAN Kit (MP Biomedicals, Cat. No. SKU 111001200). Bacterial genomic DNA was prepared by using a previously described protocol [12]. Plasmids were purified from overnight *E. coli* cultures by using Wizard *Plus* SV Minipreps DNA Purification System (Promega, Cat. No. A1460).

### PCR amplifications

PCR products were sized and isolated using agarose gel electrophoresis, cloned using the pCR2.1-TOPO TA cloning kit (Thermo Fisher Scientific, Cat. No. 450641), and transformed into chemically competent *E. coli* TOP10 or *E. cloni*® 10 G. PCR amplifications were performed in a final reaction volume of 50 μl containing FailSafe PCR System with 1X PreMix D (Lucigen, Cat. Nos. FS99100 and FSP995D-INCL), 1.25 U FailSafe PCR Enzyme Mix, 1 μM PCR primers and approximately 200 ng of genomic DNA. Colony PCR was utilized to screen for *B. pseudomallei* deletion mutants. Briefly, sucrose resistant and Km sensitive colonies were resuspended in 50 µl water and 5 µl was added to the PCR reaction rather than purified genomic DNA. PCR cycling was performed using a PTC-150 minicycler with a Hot Bonnet accessory (MJ Research, Inc.) and heated to 97°C for 5 min. This was followed by 30 cycles of a three-temperature cycling protocol (97°C for 30 s, 55°C for 30 s and 72°C for 1 min) and one cycle at 72°C for 10 min. For PCR products greater than 1 kb, an additional 1 min per kb was added to the extension time.

The primers used to PCR-amplify *BPSS1996* (*BURPS668_A2871*) were 1996-up (5ʹ-GCTAGCCGATTCGATCCTCGTGCAAC-3) and 1996-dn (5ʹ-GCTAGCAGATGCTCAATCGCAAGCTG-3ʹ). *BPSL2748* (*BURPS668_3186*) was PCR-amplified with 2748-up (5ʹ-GCTAGCGTGAACACGTACGAAGGTAC-3ʹ) and 2748-dn (5ʹ-GCTAGCACTTGTCCGGATGCGCGATG-3ʹ).

### *Production and purification of recombinant* Burkholderia *proteins*

*BPSS1996* and *hcp1* (hemolysin-coregulated protein 1) [13] were synthesized, cloned, expressed and the encoded proteins were purified commercially (Biomatik). The genes were synthesized with encoded C-terminal 6X His-tags and flanking enzyme restriction sites. Primers were designed and used to clone the PCR products into the *Nde*I and *Hin*dIII sites of plasmid pUC57 (GenBank Accession Y14837.1). *E. coli* clones harboring the target sequence were identified by blue/white screening. Recombinant plasmid DNA was extracted from the clones and Sanger sequencing done to confirm the plasmid construct. Expression and amplification of the recombinant proteins were performed by the supplier using standard procedures, and the proteins were purified on nickel Ni^2+^ His-tag affinity columns. The final proteins were shown to be >90% pure by Coomassie blue-stained SDS-PAGE and western blot using an anti-6X His antibody.

### *Polyacrylamide gel electrophoresis (PAGE) of purified* Burkholderia *proteins*

Purified proteins were adjusted to 100 µg/ml and combined with Novex™ Tricine SDS Sample Buffer (Thermo Fisher Scientific, Cat. No. LC1676) before loading the wells of a Novex™ 16% Tricine Protein Gel (Thermo Fisher Scientific, Cat. No. EC6695BOX); the lanes contained 1.0–1.5 µg protein each. A lane with Novex™ Sharp Pre-stained Protein Standard (Thermo Fisher Scientific, Cat. No. LC5800) was included for size estimations. The gel was electrophoresed and stained with SimplyBlue™ SafeStain (Thermo Fisher Scientific, Cat. No. LC6060).

### *Detection of serum antibodies to BPSS1996 and Hcp1 in a* B. pseudomallei*-infected rhesus macaque*

Enzyme-linked immunosorbent assay (ELISA) was performed by coating microtiter plate wells with 25 µg/ml of recombinant BPSS1996 and Hcp1. Irradiation-inactivated *B. pseudomallei* K96243 (BPK) [14] served as a positive control and buffer as a negative control. The antigens were incubated with nonhuman primate (NHP) antiserum from a *B. pseudomallei* K96243-infected rhesus macaque and developed with a goat anti-rhesus IgG (H + L)-HRP secondary antibody (Southern Biotech, Cat. No. 6200–05). The NHP antiserum had an anti-BPK reciprocal titer of 12,150,000 by ELISA (data not shown).

### BALB/c mouse challenge studies

Bacterial strains were grown overnight in LB broth, serially diluted in PBS, and aliquots were spread onto LB agar plates to determine the number of colony forming units (CFU) present. Six- to eight-week-old female BALB/c mice, 10 per group, were challenged by the intraperitoneal (i.p.) route with 10^1^–10^4^ CFU of *B. pseudomallei* MSHR668, 668 Δ*S1996*, and 668 Δ*L2748*. The animals were observed at least once daily and moribund animals were euthanized by CO_2_ exposure. On day 21, the surviving animals from each group were euthanized with CO_2_. A Bayesian probit analysis was performed for each strain to estimate the lethal dose response curve and the 50% lethal dose (LD_50_). Prior distributions for each parameter were assumed to be independent, weakly informative Cauchy distributions with center 0 and scale 10. Using samples from the posterior distributions of the slope and intercept parameters from the probit analysis, the median and 95% credible intervals of the range of dose responses were estimated.

Research was conducted under an IACUC approved protocol in compliance with the Animal Welfare Act, PHS Policy, and other Federal statutes and regulations relating to animals and experiments involving animals. The facility where this research was conducted, USAMRIID, is accredited by the Association for Assessment and Accreditation of Laboratory Animal Care, International and adheres to principles stated in the Guide for the Care and Use of Laboratory Animals, National Research Council, 2011.

### *Immunization of BALB/c mice with BPSS1996 and Hcp1 and challenge with* B. pseudomallei *K96243*

BALB/c mice were immunized with a prime dose of protein solution and boosted twice, at day 21 and again at day 35 after the prime dose. The doses each contained 25 µg of purified protein (BPSS1996 or Hcp1) combined with 500 µg Alhydrogel (500 µg), 20 µg deoxyoligonucleotide CpG (ODN2006) (InvivoGen, Cat. No. tlrl-2006-5), and PBS in a total volume of 200 µl. Control mice received the Alhydrogel/ODN2006 solution in PBS alone. One group was administered a solution containing a combination of both proteins. Each animal group consisted of sixteen mice, six of which were euthanized just prior to the challenge date for collection of polyclonal antisera to BPSS1996 and Hcp1. The remaining ten mice were challenged via the i.p. route 30 days after the last vaccine dose with an equivalent to 5–10 LD_50_s of *B. pseudomallei* strain K96243. Endpoint antibody titers of the sera were determined by ELISA as described above.

### Immunofluorescence microscopy

Bacteria were grown overnight in LB media with and without antibiotics. The cells were centrifuged (4000 g, 5 min), washed twice with PBS and resuspended in 750 µl of 4% paraformaldehyde for 15 min at room temperature to fix. The cells were washed three times with nanopure water containing 1% BSA and 30 µl aliquots were added to poly-L-lysine coated slides and allowed to air dry. Fifty microliters of a 1:1000 dilution of mouse polyclonal BPSS1996 antisera in 2% BSA was added to the cells and incubated for 1 h at room temperature. The slides were washed with 1% BSA and incubated with a 1:1000 dilution of Alexa®Fluor 488–tagged goat anti-mouse IgG (H + L) antibody (Invitrogen, Cat. No. A11001) for 30 min. The slides were washed with 1% BSA, allowed to air dry and a drop of mounting media was added. The slides were viewed using a Nikon eclipse 90i microscope with phase contrast (100x, oil immersion objective) and fluorescence microscopy.

### *Construction and complementation of* B. pseudomallei *mutants*

Gene replacement experiments with *B. pseudomallei* were performed using the *sacB*-based vector pMo130, as previously described [15–17]. The 1043-bp 2748-up/2748-dn PCR product was cloned into pCR2.1-TOPO (pCR2.1-*BPSL2748*), digested with *Eco*47III and *Sal*I and the ends were blunt-ended and ligated. The resulting plasmid, pCR2.1-*∆BPSL2748*, contains a 359-bp deletion of *BPSL2748*. The insert was released with *Nhe*I and cloned into the corresponding site of pMo130 and designated pMo130-*∆BPSL2748*. The 1370-bp 1996-up/1996-dn PCR product was cloned into pCR2.1-TOPO (pCR2.1-*BPSS1996*), digested with *Cla*I and *Bsi*WI and the ends were blunt-ended and ligated. The resulting plasmid, pCR2.1-*∆BPSS1996*, contains a 341-bp deletion of *BPSS1996*. The insert was released with *Nhe*I and cloned into the corresponding site of pMo130 and designated pMo130-*∆BPSS1996*. The *Nhe*I insert from pCR2.1-*BPSS1996* was also cloned into the corresponding site of pMo130, resulting in pMo130-*BPSS1996*. This construct was linearized with *Bsi*WI (14-bp upstream of the *BPSS1996* stop codon), blunt-ended with the End-It DNA End-Repair Kit and treated with Antarctic Phosphatase. A promoter-less green fluorescent protein (GFP) gene was generated by digesting pCR2.1-GFP [18] with *Bam*HI and *Xba*I and treating with the End-It DNA End-Repair Kit. The blunt-ended and promoter-less GFP gene was cloned into the repaired *Bsi*WI site of pMo130-*BPSS1996* so that the *BPSS1996* and GFP genes were in the same orientation (pS1996-GFP). The recombinant derivatives of pMo130 were electroporated (12.25 kV/cm) into *E. coli* S17-1 [19] and conjugated with *B. pseudomallei* MSHR668 and Bp82 for 8 h. Pm was used to counterselect *E. coli* S17-1. Optimal conditions for resolution of the *sacB* constructs were found to be LB agar lacking NaCl and containing 10% (w/v) sucrose, with incubation at 25°C for 4 days. *B. pseudomallei* deletion mutants were identified by colony PCR using the primers described above. The PCR products generated from the deletion mutant strains were smaller than those obtained from the wild-type strain, but the *BPSS1996*-GFP allele resulted in a larger PCR product than *BPSS1996*.

The *BPSS1996* insert was released from pCR2.1-*BPSS1996* with *Eco*RI and *Sal*I and cloned into the corresponding sites of the broad-host-range plasmid pBHR2 [18], creating pBHR2-*BPSS1996*. The purified plasmid was electroporated into Bp82 *∆BPSS1996*. Briefly, the organism was grown overnight in 5 ml of LB supplemented with adenine and thiamine. The culture was centrifuged, the pellet was washed twice with 3 ml of ice-cold 10% glycerol and resuspended in 1 ml of 10% glycerol. Approximately 1 μg of pBHR2 and pBHR2-*BPSS1996* were mixed with 40 μl of Bp82 *ΔBPSS1996* and incubated on ice for 15 mins. The mixtures were transferred to 2 mm diameter cuvettes (Thermo Fisher Scientific, Cat. No. BTX620) and electroporated for 4 ms, 2.5kv/cm and a constant capacitance of 25μF. The electroporated mixtures were immediately resuspended in 1 ml of 50% recovery media (Lucigen, Cat. No. F98226-1) and 50% LB supplemented with adenine and thiamine, transferred to 15 ml snap-cap polypropylene tubes and incubated at 37°C for 4 hours with constant agitation (250 rpm). One hundred microliters of the mixture was spread onto LB agar plates containing adenine, thiamine and Km and incubated for three days at 37°C.

### Gentamicin protection assays in RAW264.7 cells

**Figure 1.**
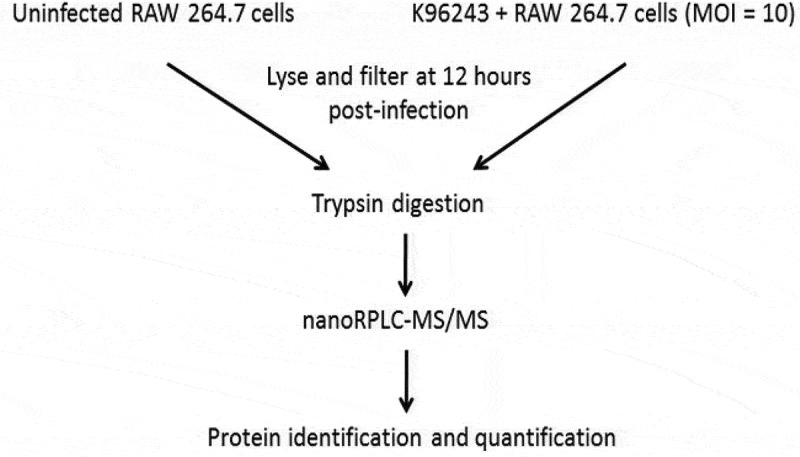
Workflow diagram of the cell culture and proteomics procedures that were used in this study. RAW264.7 macrophages and macrophages infected with *B. pseudomallei* K96243 at a MOI of 10 were lysed and filtered at 12 hours after infection. The resulting protein samples were digested with trypsin, subject to nanoRPLC-MS/MS, identified and quantified.

RAW264.7 cells were grown at 37°C in the presence of 5% CO_2_ in Dulbecco’s Modified Eagle Medium with 10% (v/v) fetal bovine serum (DMEM-10). The cells were infected with *B. pseudomallei* at a multiplicity of infection (MOI) of 10 for 1.5 h at 37°C, 5% CO_2_ to allow bacterial uptake. The cell culture media was removed and the cells were washed twice with Hanks’ Balanced Salt Solution (HBSS). DMEM-10 containing 200 μg/ml gentamicin was added to the cells and incubation was continued for an additional 10–24 h depending on the assay. For the LC-MS/MS studies (Figure 1), the media was removed at 12 h post-infection and the RAW cells were washed with PBS. The RAW264.7 cells were then lysed with 0.1% (v/v) triton X-100 in water, filtered to remove bacteria, and the lysates frozen at −70°C until they could be processed. Uninfected RAW264.7 cells served as a control for this experiment. For the intracellular replication and survival studies (Figure 5), at 1.5, 6, 9, 12, and 24 h post-infection, the RAW264.7 cells were washed, lysed and intracellular bacterial numbers were quantitated by spreading serial dilutions of the lysates on LB plates and incubating at 37◦C for 24–48 h. The experiment was performed in triplicate and the numerical values represent the mean ± S.D.

### Statistical analyses

Significant protein changes identified with MaxQuant were determined using a two sample t-test; protein intensity replicates for the two samples were grouped and the statistical test was performed with an FDR value (expected proportion of false positives) of 0.001. The results of the ELISA assays for serum antibodies were evaluated by a two-way analysis of variance (ANOVA) with posthoc Tukey’s multiple pairwise comparisons tests, as performed using Prism GraphPad version 8.4.2. A Bayesian probit analysis was performed for each strain to estimate the lethal dose response curve and the LD_50_. Prior distributions for each parameter were assumed to be independent, weakly informative Cauchy distributions with center 0 and scale 10. Using samples from the posterior distributions of the slope and intercept parameters from the probit analysis, the median (and 95% credible intervals of the range of dose responses), and the probabilities of significant differences in LD_50_ were estimated. The viable count data obtained in the cell culture assay were evaluated with the Welch’s (unequal variance) t-test using the mean log_10_-transformed CFU/ml for each time-point.

## Results

### *One hundred sixty* B. pseudomallei *proteins identified inside of RAW264.7 cells at 12 hours post-infection*

Proteomics is a powerful tool for the study of proteins in complex mixtures including cells, body fluids, tissues, organisms and other complicated samples [20]. Proteome analysis provides unique information about the modulation of host proteins during infection and can be used to identify bacterial proteins produced during infection. In this study, we utilized a proteomics approach to better understand the *Burkholderia*-macrophage interaction at the molecular level. Previous studies have shown that *B. pseudomallei* is present in the host cell cytosol in relatively large numbers by 12 h post-infection [13] and we chose this time point for our proteomic analysis. RAW264.7 cells were infected with *B. pseudomallei* K96243 for 12 h and then washed, lysed and the lysate was filter-sterilized to remove intact bacteria (Figure 1). The bacterial and host proteins were digested with trypsin and analyzed using nanoRPLC-MS/MS. One hundred sixty K96243 proteins were identified inside RAW264.7 cells at 12 h post-infection (Table 1 and S1), including some previously known to be produced only inside host cells (Hcp1, TssA, TssB, and BimA) [13,21–24]. This result indicated that our methodology was technically sound and working as predicted. Table 1 also shows some of the other *B. pseudomallei* K96243 proteins found inside RAW264.7 cells at 12 h post-infection. TssM, a known *B. pseudomallei* secreted effector protein with an N-terminal signal sequence, was among the proteins identified [15,24]. While our initial goal was to enrich for bacterial proteins secreted inside macrophages, many cytoplasmic and cell-associated bacterial proteins were also released inside the host cells by the inherent bactericidal activity of the RAW264.7 cells (Table 1 and S1). This result suggests that not all bacteria are fully intact at 12 h post-infection in RAW264.7 cells and the bacterial proteins in Table 1 and S1 more accurately represent the cytoplasmic, cell-associated and secreted proteins that are abundantly produced when *B. pseudomallei* is grown intracellularly.

**Table 1.** Selected *B. pseudomallei* K96243 proteins identified inside RAW264.7 macrophages at 12 hours post-infection.

Locus tag (gene)	Protein description	Peptide counts^a^
BPSL2697 (*groEL*)	60 kDa chaperonin GroEL	92
BPSL3215 (*tuf*)	Elongation factor EF-Tu	66
BPSL3216 (*fusA*)	Elongation factor EF-G	29
BPSS1498 (*hcp1*)	Type VI secretion system 1 (T6SS-1) tail tube protein Hcp1	25
BPSL2698 (*groES*)	10 kDa chaperonin GroES	22
BPSL1087 (*htpG*)	Chaperone protein HtpG	18
BPSL2827 (*dnaK*)	Chaperone protein DnaK	16
BPSL2829 (*grpE*)	Heat shock protein GrpE	11
BPSL2748	Peroxiredoxin, AhpC-type	10
BPSL2158 (*tsf*)	Elongation factor EF-Ts	9
BPSL1929	Putative exported protein; hypothetical	7
BPSS1996	Putative exported protein; DUF4148 protein	4
BPSL0880 (*sodB*)	Superoxide dismutase SodB; FeSOD	4
BPSL0999	Outer membrane protein OmpA and related peptidoglycan-associated (lipo)proteins	4
BPSL2765	Outer membrane protein OmpA and related peptidoglycan-associated (lipo)proteins	4
BPSS1497 (*tssB*)^b^	T6SS-1 protein TssB	3
BPSS1512 (*tssM*)^b^	Secreted deubiquitinase TssM	3
BPSS0032 (*uspA*)	Universal stress protein A UspA	3
BPSS1492 (*bimA*)	*Burkholderia* intracellular motility A protein BimA	2
BPSL2522 (*ompA*)	Outer membrane protein OmpA and related peptidoglycan-associated (lipo)proteins	2
BPSL2520	Putative exported protein; DUF2059 protein	2
BPSL1933 (*bamC*)	Outer membrane protein assembly factor BamC	2
BPSS1496 (*tssA*)^b^	T6SS-1 protein TssA	2
BPSL3146 (*mlaC*)	Mla ABC transport system	1
BPSL1418	Putative exported Peptidyl-prolyl cis-trans isomerase	1

^a^Peptide counts are the number of unique peptides identified by nanoRPLC-MS/MS using MaxQuant software.

^b^The *B. pseudomallei* TssA and TssB proteins are the T6SS-1 sheath proteins that are often referred to as TssB and TssC in other Proteobacteria. TssA is a T6SS capping protein and TssM is a component of the membrane complex in the alternative T6SS nomenclature used in other bacteria.

The *B. pseudomallei* proteins detected within RAW264.7 cells included those encoding stress-associated proteins (heat shock proteins and detoxifying enzymes), outer membrane proteins (OMPs), putative exported proteins and cytoplasmic elongation factors (Table 1 and S1). The bacterial heat shock proteins (HSPs) detected inside macrophages included the chaperones GroES, GroEL, HtpG, DnaK and GrpE (Table 1). These proteins are highly conserved proteins but have been shown to have important roles in infection, and in the immune response to and diagnosis of infection [25–33]. Additional intracellularly produced bacterial proteins associated with host-mediated nutritional and oxidative stress included the universal stress protein A (UspA), peroxiredoxin BPSL2748 and superoxide dismutase BPSL0880 (SodB). Three of the twelve OmpA family proteins encoded in the *B. pseudomallei* K96243 genome, BPSL0999, BPSL2765 and BPSL2522, were also detected inside RAW264.7 macrophages (Table 1) [34]. These proteins interact with peptidoglycan to maintain membrane and cell morphology and are known to be involved in bacterial virulence [35,36]. Another *B. pseudomallei* OMP induced inside macrophages, BamC, is part of the OMP assembly complex (BAM) involved in assembly and insertion of beta-barrel proteins into the outer membrane. It stabilizes the interaction between the essential proteins BamA and BamD [37,38]. Several of the proteins in Table 1 are predicted to be putative exported proteins because they harbor N-terminal signal sequences, including the secreted effector TssM [15,24]. Finally, the cytoplasmic elongation factors EF-Tu, EF-Ts, and EF-G, which are essential to the process of translating RNA into proteins, were also identified inside the infected macrophage cells (Table 1 and S1). This result indicates that there was likely appreciable bacterial lysis in RAW264.7 cells by 12 h post-infection and some of the proteins in Table 1 and S1 represent integral cytosolic bacterial proteins abundantly produced in the intracellular niche. The overall *Burkholderia* expression profile in cultured macrophages appears to resemble the *B. pseudomallei* gene expression pattern observed in human patients, as discussed below.

### Two hundred seventy-four host proteins were exclusively present or absent in infected RAW264.7 cells at 12 h post-infection

We predict that *B. pseudomallei* growth inside macrophages will modulate the production of numerous components of the innate immune system and signaling pathways involved in the pathogenesis of infection. The proteomic analysis of uninfected and infected RAW264.7 cells yielded 274 host proteins that were exclusively present (58) or absent (216) in the infected cells (Table 2 and S2). Furthermore, there were >100 macrophage proteins that exhibited at least a 10-fold difference under these two experimental conditions (data not shown). Among the 15 host proteins most impacted by *B. pseudomallei* K96243 infection, nine were up-regulated and six were down-regulated compared to the protein levels in uninfected cells (Table 2). Two of the three most highly upregulated proteins were the cytokines interleukin 1α (IL-1α) and 1β (IL-1β), proinflammatory cytokines having pleiotropic activities. They play a central role in regulation of the immune response and inflammation and directly activate NF-κB, a global inflammatory response regulator. Several other cytokines/chemokines or their receptors displayed increased abundance, i.e., chemokine CXCL2 and C-C receptor CCR1 and granulocyte colony stimulating factor. G-CSF stimulates the bone marrow to produce polymorphonuclear leukocytes and their precursor stem cells and thus has an important role in the inflammatory response to infection. Similarly, elevated levels of complement protein C3, the most abundant protein of the complement system, signals a stimulated host response to *B. pseudomallei* infection.

**Table 2.** RAW 264.7 proteins exclusively present or absent inside *Burkholderia*-infected cells at 12 hours post-infection.

Gene	Protein	Uninfected LFQ intensity^a^	Infected LFQ Intensity
*Il1b*	Interleukin 1β (IL-1β)	0	1.8 x 10^9^
*Il1a*	Interleukin 1α (IL-1α)	0	2.9 x 10^8^
*Cxcl2*	C-X-C motif chemokine ligand 2 (CXCL2)	0	3.7 x 10^8^
*Tnfrsf1b*	Tumor necrosis factor superfamily member 1B (TNFRSF1B)	0	9.6 x 10^7^
*Cd40*	Tumor necrosis factor receptor superfamily member 5 (CD40)	0	8.3 x 10^7^
*Ccr1*	C-C chemokine receptor 1 (CCR1)	0	3.6 x 10^7^
*Csf3*	Granulocyte colony-stimulating factor (G-CSF)	0	2.4 x 10^7^
*Traf1*	TNF receptor-associated factor 1 (TRAF1)	0	8.5 x 10^6^
*C3*	Complement C3	0	2.1 x 10^6^
*Gps1*	COP9 signalosome complex subunit 1	3.9 x 10^7^	0
*Nkrf*	NF-κB -repressing factor (NKRF)	9.3 x 10^6^	0
*Irak1*	Interleukin-1 receptor-associated kinase 1 (IRAK1)	7.2 x 10^6^	0
*Ap3b2*	AP-3 complex subunit beta-2	6.4 x 10^6^	0
*Casp6*	Caspase-6	5.1 x 10^6^	0
*Card9*	Caspase recruitment domain-containing protein 9 (CARD9)	4.9 x 10^6^	0

^a^LFQ, Label-free quantification

There were also significant changes in proteins involved in apoptotic cell-death functions. Three of the upregulated proteins (CD40, TNFRSF1B and TRAF1) are involved in the expression of tumor necrosis factor-alpha (TNF-α), a major stimulator of apoptosis. Nevertheless, the pleiotrophic TNF-α is also an important proinflammatory cytokine produced in higher abundance in individuals with melioidosis as compared with uninfected healthy individuals [39]. Furthermore the only proteins typically associated with apoptosis and which were notably altered in expression were the enzyme caspase 6 and the caspase recruitment domain family protein CARD9. They were down-regulated. Finally, the six down-regulated proteins (Table 2) also include entities involved in the down-regulation or reduced expression of the transcriptional activator NF-κB. Thus, all of the host proteins identified in Table 2 as being strongly affected by infection may play important roles in the pathogenesis of *B. pseudomallei* in the host.

### *Construction of deletion mutations in two* B. pseudomallei *genes expressed in RAW267.4 cells*

**Figure 2.**
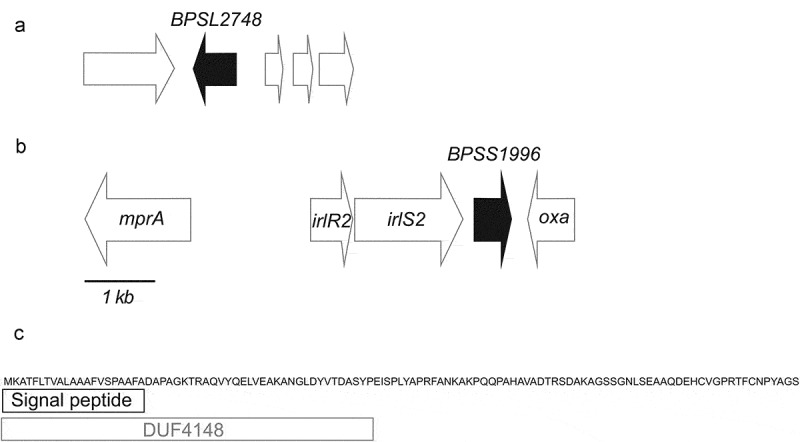
Genomic context of the genes analyzed in this study and the domain organization of BPSS1996. **a**) *B. pseudomallei* K96243 chromosome 1 genes *BPSL2747-BPSL2751* are shown as arrows and *BPSL2748*, a gene encoding a putative peroxiredoxin, AhpC-type, is shaded. **b**) *B. pseudomallei* K96243 chromosome 2 genes *BPSS1993-BPSS1997* are shown as arrows and *BPSS1996*, a gene encoding a DUF4148 protein, is shaded. *BPSS1993* (*mprA*) encodes metalloprotease A, a serine protease of the subtilisin family [75]. *BPSS1994* (*irlR2*) and *BPSS1995* (*irlS2*) encode a two-component regulatory system and exhibit > 70% nucleotide identity with the *B. pseudomallei* 1026b invasion-related locus genes *irlR* and *irlS* (33). *BPSS1997* (*oxa*) encodes a class D β-lactamase [76]. A 1 kb scale for A) and B) is shown at the bottom of B). Open spaces between the genes represent non-coding intergenic regions. **c**) Domain organization of the 113 amino acid protein BPSS1996. The N-terminal 53 amino acids of BPSS1996 form a DUF4148 superfamily domain, which includes a 21 amino acid signal peptide [77].** The C-terminal 60 amino acids do not share sequence similarity with any of the currently recognized protein domain families.

Bacterial proteins produced inside of host cells represent potential virulence determinants and several of the *B. pseudomallei* proteins we identified inside RAW264.7 cells are known virulence factors, including Hcp1, BimA and TssM (Table 1). Many of the K96243 proteins we identified in RAW264.7 at the 12 h time point are proteins that are novel or have no defined function. We selected two K96243 genes, *BPSS1996* and *BPSL2748* (Table 1 and Figure 2), for mutagenesis in the heterologous strain MSHR668 [9]. This *B. pseudomallei* strain was selected for further studies because it is highly virulent for BALB/c mice by the i.p. route of infection (see below). Our K96243 isolate, on the other hand, is noticeably less virulent for BALB/c mice by the i.p. route of infection [40]. BPSL2748 is predicted to be a peroxiredoxin, an antioxidant enzyme that uses thioredoxin to recharge after reducing hydrogen peroxide to water. The gene encoding this protein appears to be monocistronic (Figure 2a). BPSS1996 is a novel protein with a signal peptide and a Domain of Unknown Function designated DUF4148 (Figure 2b,c). The gene encoding the DUF4148 protein is present immediately downstream of a predicted two-component signal transduction regulatory system (TCS) and may be part of a three gene operon (Figure 2b). The upstream TCS, BPSS1994-BPSS1995 (IrlR2-IrlS2), most closely resembles the *B. pseudomallei* IrlRS TCS that regulates genes involved in resistance to Cd^2+^ and Zn^2+^ [41]. In fact, IrlR2 is 73% identical to IrlR and IrlS is 50% identical to IrlS [41]. It is currently unknown if IrlR2S2 TCS is also involved in regulating resistance to heavy metals. Interestingly, a different DUF4148 gene (*BPSS1038*) is present immediately downstream of the *irlR* and *irlS* genes. No previous studies have identified any interaction of DUF4148 proteins with bacterial TCS and the close relationship of these genes in these two clusters may be coincidental.

### B. pseudomallei *668* ΔS1996 *is highly attenuated in BALB/c mice*

**Figure 3.**
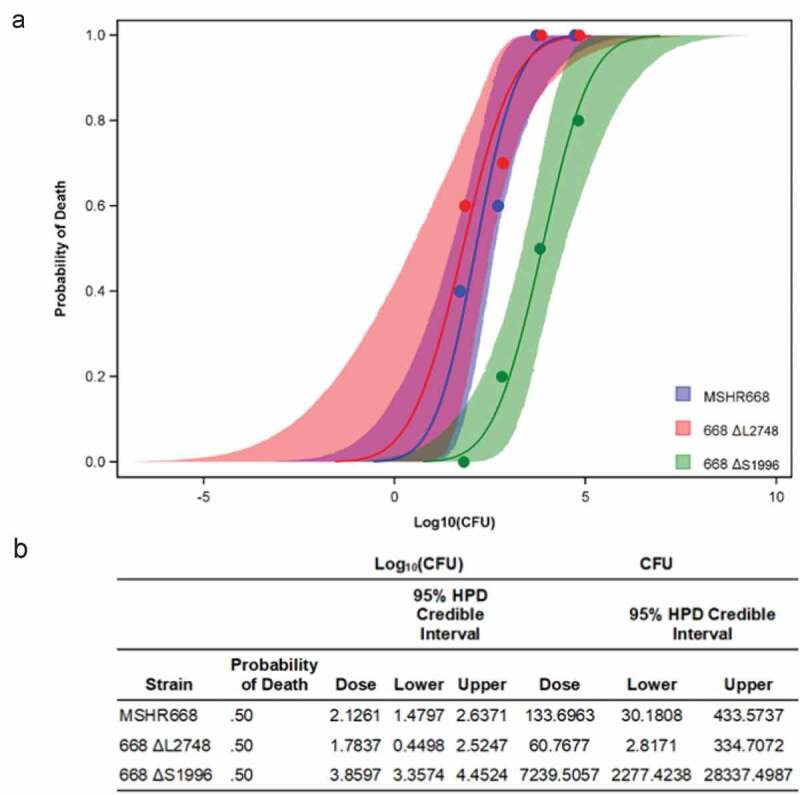
Survival curves of mice infected with *B. pseudomallei* strains MSHR668, 668 Δ*L2748* or 668 Δ*S1996*. a) Dose-related probabilities of death; b) Final calculated LD_50_s for MSHR668 (134 CFU), 668 Δ*L2748* (61 CFU) and 668 Δ*S1996* (7240 CFU) and the corresponding 95% credible limits.

In order to determine the relative virulence of the mutant strains, we infected BALB/c mice by the i.p. route with 10, 100, 1,000, and 10,000 CFU and monitored them for 21 days. *B. pseudomallei* 668 *∆S1996 (∆BPSS1996*) was highly attenuated in BALB/c mice at all doses examined, but there was no significant difference in the virulence of 668 *∆L2748* (*∆BPSL2748*) and MSHR668 (Figure 3 and S1). The 50% lethal dose (LD_50_) for 668 *∆S1996* in BALB/c mice was 7,240 CFU, ~ 55-fold higher than the LD_50_ for MSHR668 (134 CFU, p = 0.01). Our results demonstrate that BPSS1996 is an important *B. pseudomallei* virulence factor. BPSL2748, on the other hand, is not required for virulence in this animal model of infection. It was not attenuated in virulence and had an LD_50_ of 61 CFU, not significantly different from that of strain 668 (Figure 3 and S1).

### B. pseudomallei *668* ΔS1996 *does not exhibit a growth deficiency in rich media or inside RAW264.7 cells*

**Figure 4.**
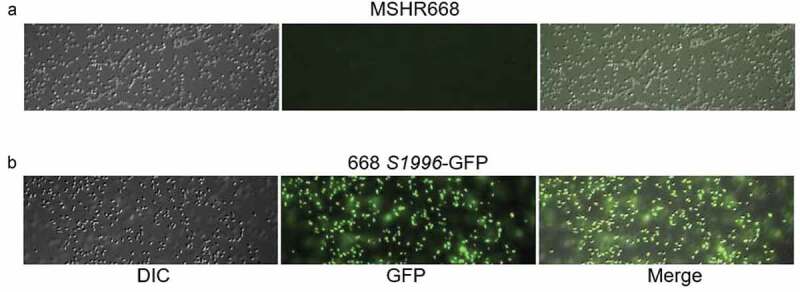
A *BPSS1996*-GFP transcriptional fusion is expressed when *B. pseudomallei* is grown in rich medium. a) MSHR668 and b) 668 *S1996*-GFP were grown in LB broth for 18 h, washed with PBS and viewed with a Nikon Eclipse 90i Fluorescent Microscope using the 100x oil immersion objective. The left panels show bacteria by differential interference contrast (DIC), the middle panels show green fluorescent protein (GFP) expression and the right panels present a merged image of the DIC and GFP images.

We next examined the growth of 668 *ΔS1996* in LB broth to ensure that the virulence deficiency of this strain was not simply due to a growth defect. The growth of this strain and the parental strain, MSHR668, were indistinguishable in LB broth (Fig. S2). In addition, we constructed a single-copy *BPSS1996*-GFP transcriptional fusion on the chromosome to examine the expression of *BPSS1996* in rich broth. Figure 4 shows that the strain possessing the *BPSS1996*-GFP allele, 668 *S1996*-GFP, exhibits green fluorescence when grown in LB broth, but MSHR668 does not. The results demonstrate that while *BPSS1996* is expressed during growth in rich broth, the gene is not required for optimal growth in this medium (Figure 4 and S2).

**Figure 5.**
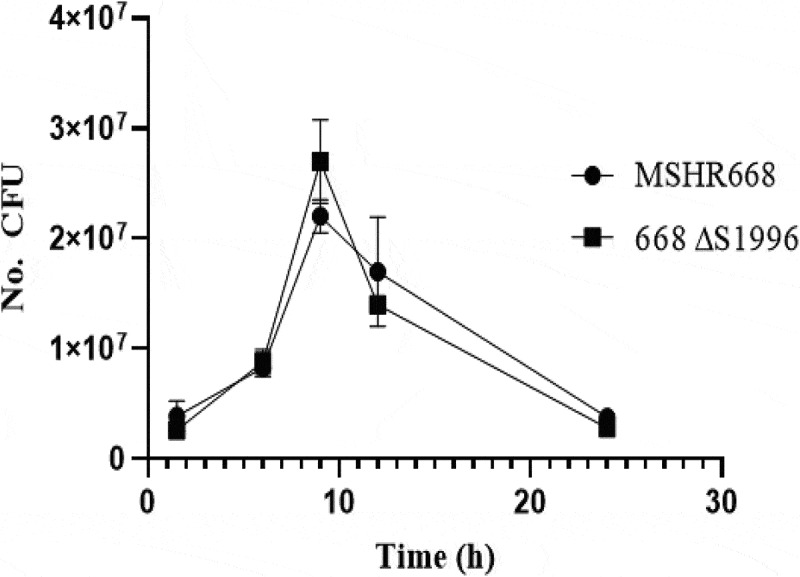
Gentamicin protection assay in RAW264.7 cells. The cells were infected at a multiplicity of infection of ~10 with *B. pseudomallei* MSHR668 and 668 *∆S1996*. At 1.5, 6, 9, 12, and 24 hours post-infection, the RAW264.7 cells were lysed and intracellular bacterial numbers were quantitated by spreading serial dilutions of the lysates on LB plates and incubating at 37◦C for 24–48 hours. The experiment was performed in triplicate and the numerical values represent the mean ± the standard deviation.

Since the BPSS1996 protein was identified inside K96243-infected macrophages (Table 1), we next examined if a mutation in the gene encoding this protein had an effect on the ability of *B. pseudomallei* to survive and replicate inside of macrophages. A gentamicin protection assay was performed to examine the ability of 668 *∆S1996* to survive and replicate inside RAW264.7 cells. Figure 5 demonstrates that 668 *∆S1996* does not display a survival or replication phenotype in RAW264.7 cells and suggests that the virulence phenotype of this strain in mice is unrelated to survival and replication in macrophages *in vivo*. The mean recovery of viable bacteria of the mutant and wild type strain at each of five time-points after infection did not differ significantly (p = 0.17, 0.57, 0.10, 0.40, and 0.10, respectively (Figure 5).

### *BPSS1996 is immunogenic in a rhesus macaque and BALB/c mice, but does not confer protection against a lethal* B. pseudomallei *challenge*

**Figure 6.**
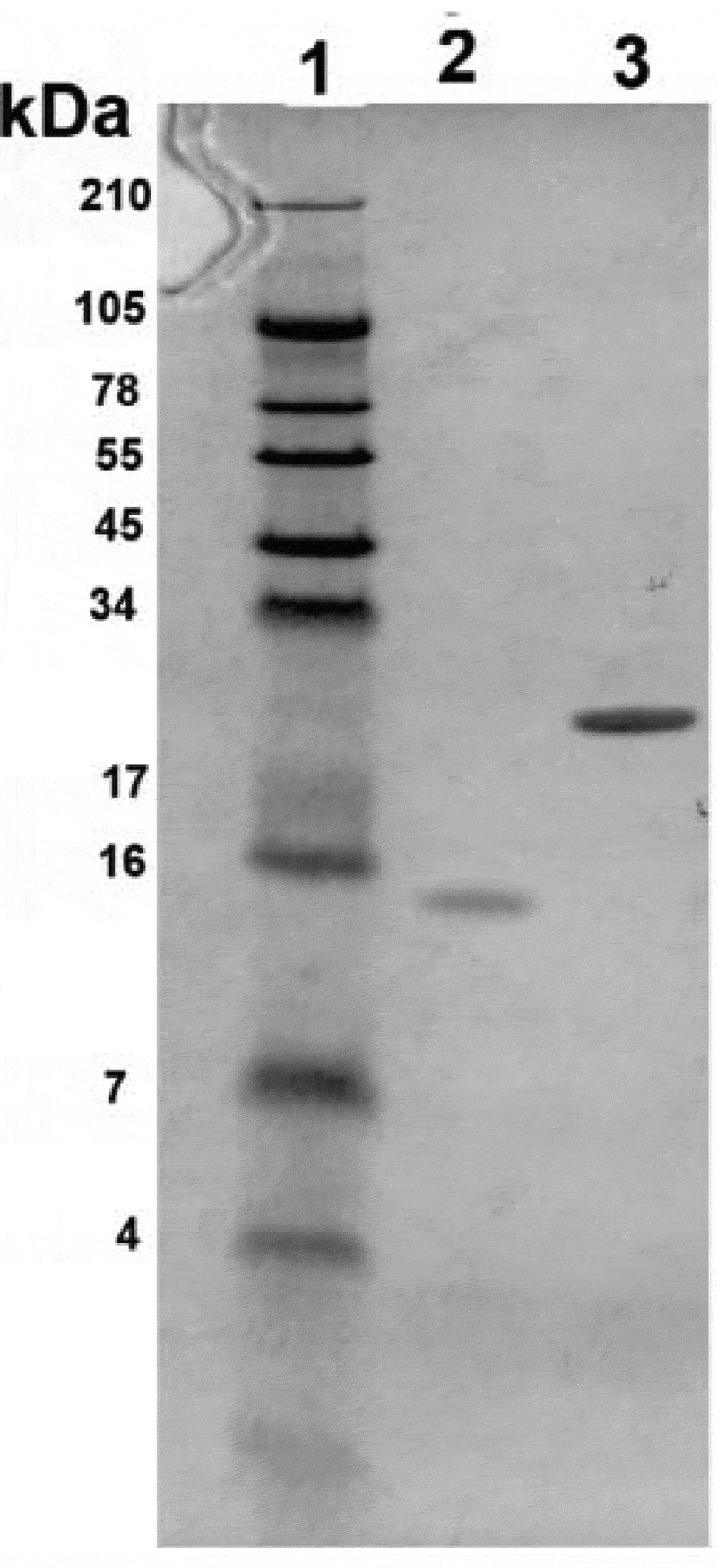
Polyacrylamide gel electrophoresis of *Burkholderia pseudomallei* proteins. Purified proteins Hcp1 and BPSS1996 were electrophoresed to confirm their purity, mobility and size. The gel was run and stained with Coomassie blue and the samples were adjusted to 100 µg/ml before loading the wells. The bands sizes agreed with the predicted sizes of 12 kDa for BPSS1996 and 22 kDa for Hcp1. Lanes (left to right): 1, Protein ladder; 2, BPSS1996; 3, Hcp1. Protein Molecular Weight (http://www.cellbiol.com/sequence_manipulation_suite/protein_mw.php) was utilized to predict the molecular weights of Hcp1 and BPSS1996 using the corresponding amino acid sequences.

**Figure 7.**
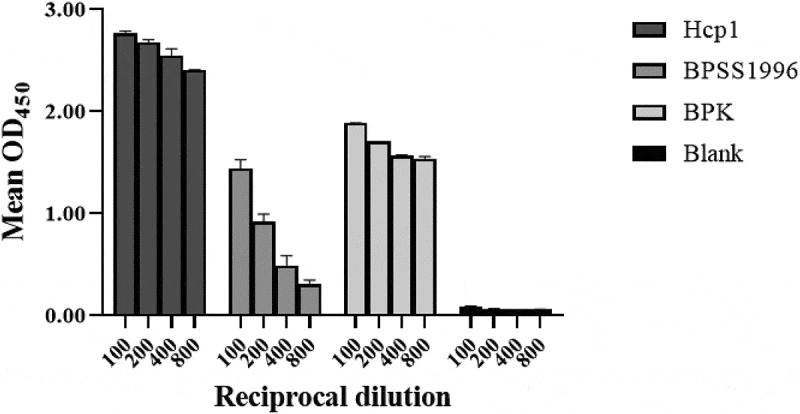
Immunoreactivity of Hcp1 and BPSS1996 with antiserum from a rhesus macaque infected with *B. pseudomallei* by ELISA. Microtiter plate wells were coated with 25 µg/ml of the antigens being tested (left to right): Hcp1, BPSS1996, killed whole cell *B. pseudomallei* strain K96243 (BPK) as positive control, or buffer alone. The control wells (blank) were incubated with buffer alone in the absence of an antigen. The proteins were detected with anti-*Burkholderia* antiserum from a rhesus macaque infected with *B. pseudomallei* strain K96243 as the primary antibody and developed with HRP conjugated goat-anti-monkey secondary antibody. All antigen-antibody samples were assayed in duplicate and the data were significantly different by ANOVA performed as described in the Materials and Methods. All 24 pairwise comparisons by dilution were significantly different with p < 0.0001 except for comparison of the reciprocal dilution 800 values of BPSS1996 and the blank sample, with the former being greater than the latter, p = 0.0002.

The genes encoding BPSS1996 and Hcp1 were synthesized, cloned, expressed and purified commercially (Biomatik), as described in the Materials and Methods. Hcp1 is a critical component of the cluster 1 type six secretion system (T6SS-1) and was included in these studies as a known immunogenic virulence-associated protein of *B. pseudomallei* [13]. To verify purity of the recombinant proteins, BPSS1996 and Hcp1 were subjected to PAGE analysis. As shown in Figure 6, the migration of the proteins on a polyacrylamide gel stained with Coomasie blue agreed with the predicted sizes of 22 kDa for Hcp1 and 12 kDa for BPSS1996. Both proteins were detected by antisera collected from a *B. pseudomallei*-infected nonhuman primate (NHP), indicating that BPSS1996 is produced *in vivo* and stimulates the host immune system (Figure 7).

BALB/c mice were immunized three times subcutaneously with Hcp1, BPSS1996 and Hcp1 + BPSS1996 in a formulation containing Alhydrogel (adjuvant) and ODN2006 (immunostimulator). Control mice were vaccinated with Alhydrogel/ODN2006 in PBS alone. Each group consisted of sixteen mice, six of which were euthanized just prior to the challenge date for serum collection and analysis. The Hcp1- and BPSS1996-immunized mice generated strong IgG responses to the recombinant proteins, with mean reciprocal endpoint titers of 4,841,700 (Hcp1) and 720,000 (BPSS1996) (Table S3). The remaining ten mice in each group were challenged one month after the last vaccine dose by the i.p. route with 6.6 × 10^5^ CFU of *B. pseudomallei* K96243 (~10 LD_50_s). All mice died by day 21 and the recombinant proteins, alone or in combination, did not extend the time to death (TTD) relative to the control group (Table 3). In fact, the TTD of the Hcp1 + BPSS1996 group was shorter than that of the mock-vaccinated group by nearly five days. Thus, the proteins alone or in combination were not protective and may possibly have exacerbated the infection and hastened the TTD compared to unvaccinated controls. These responses were not due to the absence of an immune response to the proteins as the level of IgG anti-Hcp1 and anti-BPSS1996 antibodies were substantial (Table S3). It is conceivable that the recombinant proteins induced a damaging host response referred to as a “cytokine storm”, a well-documented phenomenon which has been described in many other vaccination and infection scenarios [42]. Alternately, although primarily observed in viral infections, antibody-dependent enhancement of infection (ADE) has been described for bacterial diseases [43].

**Table 3.** Survival of BALB/c mice vaccinated with Hcp1 and/or BPSS1996 and challenged with a lethal i.p. dose of *B. pseudomallei* K96243.

Group	Vaccine	Number of dead/total^a^	Mean TTD^b^
1	PBS control	10/10	11.0
2	Hcp1	10/10	10.6
3	BPSS1996	10/10	9.8
4	Hcp1 + BPSS1996	10/10	6.2

^a^These data are the mortalities at 21 days post-challenge. The dose of K96243 was 6.6 × 10^5^ CFU (~10 LD_50_s).

^b^TTD is the mean time to death, in days

### *Localization of BPSS1996 to the surface of* B. pseudomallei

**Figure 8.**
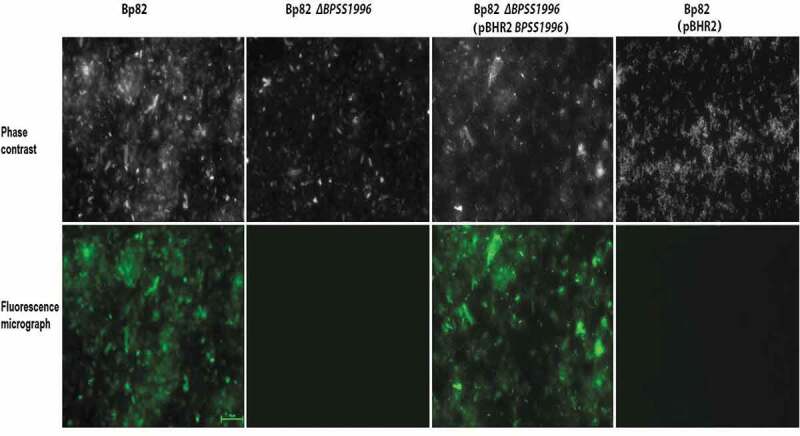
Immunofluorescence staining of bacterial cells with polyclonal anti-BPSS1996 antisera. *B. pseudomallei* Bp82, Bp82 *ΔBPSS1996*, Bp82 *ΔBPSS1996* (pBHR2-*BPSS1996*) and Bp82 *ΔBPSS1996* (pBHR2) were reacted with a murine polyclonal anti-BPSS1996 primary antibody, washed and stained with an anti-mouse Alexa 488-conjugated secondary antibody. Top panel, phase contrast; bottom panel, green fluorescence. Bar 10 µm.

Previous studies have identified BPSS1996 as an OMP [44] that elicits a humoral immune response in melioidosis patients [45]. Immunofluorescence microscopy was utilized to detect the localization of BPSS1996 protein on the surface of bacterial cells (Figure 8). Bp82, an attenuated derivative of *B. pseudomallei* 1026b [10], was incubated with polyclonal mouse anti-BPSS1996 antiserum and stained with a fluorophore 488 tagged antibody. Figure 8 shows that green fluorescence was observed with Bp82, but was absent with Bp82 *∆BPSS1996*, demonstrating that BPSSS1996 is localized on the bacterial surface. The *BPSS1996* gene was cloned into the broad-host-range plasmid pBHR2 [18] under the control of a constitutive promoter and transformed into Bp82 *∆BPSS1996*. When reacted with the anti-BPSS1996 antisera and the fluorophore 488 tagged antibody, Bp82 *∆BPSS1996* (pBHR2-*BPSS1996*) exhibited green fluorescence while the vector only control, Bp82 *∆BPSS1996* (pBHR2), did not (Figure 8). As a control, the bacteria were also incubated with BSA rather than the polyclonal BPSS1996 antisera and no florescence was detected following treatment with the secondary antibody (data not shown). The results demonstrate that the *∆BPSS1996* mutation can be complemented *in trans* and confirm that the BPSS1996 protein is present on the outer membrane of the bacterial cell.

## Discussion

NanoRPLC-MS/MS was utilized in this study to identify proteins produced by *B. pseudomallei* while inside murine macrophages. The intracellular protein profile closely resembled the gene expression of *B. pseudomallei in vivo*, especially as described for human immune responses characterized using sera from patients infected with *B. pseudomallei* [25,26,28–32,45-48]. We predicted that previously characterized *Burkholderia* proteins would be identified and serve as a positive control for our technical approach. One hundred sixty K96243 proteins were identified inside RAW264.7 cells at 12 h post-infection (Table 1 and Table S1), including some previously shown to be produced only inside host cells (Hcp1, TssM, TssA, TssB and BimA) [13,21–24]. This result provided confidence that the bacterial proteins identified were actively produced intracellularly.

Several HSPs and OMPs were present within RAW264.7 cells at 12 h post-infection, suggesting that they may enhance pathogen survival and facilitate infection in this adverse host environment. HSPs, such as GroES, GroEL, HtpG, GrpE and DnaK, are often up-regulated under conditions of environmental stress are well represented in the *B. pseudomallei* intracellular proteome (Table 1) [25,49]. These proteins are involved in the correct folding and assembly of polypeptides released from the ribosome, the transport of proteins to various locations in the cell and the degradation of aggregated or misfolded proteins. Several OMPs were also prominent in *B. pseudomallei*-infected macrophages (Table 1). As described above, BamC is a member of the BAM complex, an assembly of proteins involved in the assembly and insertion of beta-barrel proteins into the outer membrane. Although it is well recognized in *E. coli* and other bacteria [37,38], BamC was not demonstrated to be an abundant entity in previously reported *Burkholderia* proteomics studies [26,29,31,32,44,45,47] and the significance of its enhanced production in the current study requires further investigation. Three OmpA family proteins (BPSL0999, BPSL2765 and BPSL2522) were also identified inside RAW264.7 macrophages infected with K96243 (Table 1). Previous studies demonstrated that BPSL2765 and BPSL2522 were immunogenic in *B. pseudomallei*-infected mice and in melioidosis patients and provided 50% protection against a lethal challenge when used as a vaccine candidate [28,34]. It is likely that HSPs and OMPs are released inside macrophages as a result of bacterial lysis by the 12 h timepoint, but it is also possible that bacterial OMPs are shed, or proteolytically released, while growing inside macrophages.

Several *B. pseudomallei* elongation factors and antioxidant enzymes were also prevalent inside RAW264.7 macrophages. The universal elongation factors EF-Tu, EF-G and EF-Ts play major roles during the elongation cycle of protein synthesis on the ribosome and they were well-represented inside infected macrophages (Table 1 and S1). Interestingly, EF-Tu also serves as a chaperone during times of environmental stress and participates in the folding of denatured proteins [50]. The antioxidant enzymes peroxiredoxin (BPSL2748), FeSOD (BPSL0880), thiol peroxidase (BPSL2987) and AhpC (BPSL2096) were among the top K96243 proteins present inside macrophages (Table 1 and S1). These enzymes are involved in the detoxification of reactive oxygen species (ROS), such as hydrogen peroxide, alkyl hydroperoxides and superoxide, and promote the survival of pathogens exposed to antibacterial environments such as the host cell phagolysosome [51,52]. *B. pseudomallei* AhpC was found to be highly expressed in plasma from melioidosis survivors, but not in healthy controls [31]. In addition, T cell mediated immunity to AhpC was associated with protection against disease and shown to be a correlate of survival [46,48] and it represents a melioidosis vaccine candidate [30,46,48]. BPSL2748 is a putative AhpC-type peroxiredoxin, but it has not been as extensively studied as *B. pseudomallei* AhpC. In this study, we constructed a *BPSL2748* mutant, 668 *∆L2748*, and showed that it exhibited no overt decrease in virulence in a BALB/c mouse i.p. model of melioidosis (Figure 3 and S1). This result implies that *BPSL2748* may encode a redundant activity due to expression of other *B. pseudomallei* anti-oxidative enzymes (Table 1 and S1). Alternately, changes in virulence associated with the loss of BPSL2748 might be dependent on the route of infection.

The final group of *B. pseudomallei* proteins identified within infected RAW264.7 murine macrophages were putative exported proteins containing N-terminal signal sequences (Table 1). Proteins belonging to this group were BPSL1929, BPSS1996, BPSS1512 (TssM), BPSL2520 and BPSL1418. TssM is a deubiquitinase that is secreted by the *Burkholderia* type II secretion system (T2SS) [15,53]. It is induced inside of phagocytic cells where it downregulates the innate immune response by interfering with the ubiquitination of signaling intermediates involved in TLR-mediated NF-κB activation [15,24]. BPSL1929 is a hypothetical protein and BPSL1418 is a predicted peptidyl-prolyl cis-trans isomerase (PPIase), a superfamily of proteins associated with multiple biological activities and virulence [54]. BPSS1996 and BPSL2520 are members of the Domains of Unknown Function (DUF) families DUF4148 and DUF2059, respectively. DUF proteins are highly conserved, but have no known biochemical activity or structural characterization [55]. The *BPSS1996* mutant strain constructed in this study, 668 *∆S1996*, was highly attenuated in BALB/c mice infected by the i.p. route of infection (Figure 3). This finding suggests that BPSS1996 is a novel virulence factor of *B. pseudomallei*. While this protein was identified within infected RAW264.7 cells (Table 1), it was not involved in intracellular replication and/or survival in macrophages (Figure 5). This result was somewhat surprising and may indicate that BPSS1996 somehow alters the *in vivo* biology of macrophages [56], perhaps by influencing antigen presentation capacity and/or cytokine and chemokine production, and thus hindering the host’s ability to adequately eliminate the infection. BPSS1996 is produced by *B. pseudomallei in vitro* (Figure 4) and *in vivo* as it is recognized by melioidosis antisera from infected NHPs (Figure 7) and humans [45]. Schell *et al*. first characterized BPSS1996 as a *B. pseudomallei* OMP [44] and fluorescence microscopy was used here to confirm that it is surface-associated (Figure 8).

While DUF4148 proteins are found in both bacteria and eukaryotes, the overwhelming majority are present in members of the order *Burkholderiales* (Fig. S3). The *B. pseudomallei* K96243 genome encodes eight DUF4148 proteins (Fig. S4) and all the genes encoding these proteins are present on chromosome 2. Accessory genes, such as those involved in secondary metabolism, environmental survival and pathogenesis, are relatively common on chromosome 2 [6]. Two closely related *Burkholderia* species, *B. mallei* and *B. thailandensis* [8], possess five and eleven DUF4148 proteins, respectively (Fig. S4). *B. thailandensis*, an environmental saprophyte, contains a BPSS1996 ortholog (BTH_II0374), but the gene encoding this ortholog was lost by IS*407*A-mediated genome reduction in the host-adapted pathogen *B. mallei* [57] (Fig. S4). The *Burkholderia* DUF4148 proteins are annotated as exported proteins with a ~ 21 amino acid signal peptide embedded within a conserved N-terminal domain of ~ 54 amino acids (Figure 2c and S5). The *B. pseudomallei* DUF4148 proteins are relatively small, 90–113 amino acids, and contain C-terminal portions that display no similarities to proteins in the Pfam database [58]. Very little has been published in the literature on DUF4148 proteins, but a recent publication found that the parasite *Herpetomonas muscarum* possesses a putative surface-exposed DUF4148 protein that is up-regulated in its fruit fly host [59]. Further studies will be required to determine the function of the surface-exposed BPSS1996 protein and elucidate the role it plays in *B. pseudomallei* pathogenesis.

RAW264.7 proteins were also significantly impacted by the *B. pseudomallei* infection. We identified 274 host proteins that were either exclusively present (58 total) or absent (216 total) in K96243-infected cells, including a number of chemokines and cytokines. Table 2 describes the 15 macrophage proteins most impacted by *B. pseudomallei* K96243 infection, nine of which are increased and six are decreased compared to the protein levels in uninfected cells. Since *B. pseudomallei* is an intracellular pathogen, innate immune responses of the host are involved in controlling the initial stages of infection. The mostly highly expressed factors shown in Table 2 are innate immune signaling molecules including proinflammatory cytokines IL-1α, IL1-β, and G-CSF; TNF-α ligands; and chemokines. Elevated levels of these cytokines are commonly identified as major responders to *B. pseudomallei* infection in tissues from humans and mice infected with *B. pseudomallei* or stimulated with *B. pseudomallei* antigens [60–67].

In a RAW264.7 cell model of *B. pseudomallei* infection, Hseu *et al*. observed the increased production of many apoptosis-associated proteins to include several caspase enzymes and caspase-associated recruitment proteins (CARD) as well as numerous ligands of TNF, a major stimulator of apoptosis [68]. Three of the nine highly expressed entities in Table 2 are TNF receptor-associated proteins (encoded by TNFRSF1B, CD40, and TRAF1). However only one CARD family protein gene (*Card9*) and one caspase enzyme prominent in apoptosis (CASP6) are included, both of which were down-regulated (Table 2). Several other RAW264.7 proteins were down-regulated relative to uninfected cells (Table 2): IRAK1 (IL-1-receptor associated kinase), NKRF (NF-κB-repressing factor), COPS1 (COP9 signalosome complex subunit 1), and AP3B2 (AP-3 complex subunit beta-2). Since NF-κB has a central role in regulating the cellular response to infection and activates transcription of cytokines and survival functions, the down-regulation of NKRF in infected macrophages is a logical finding. Conversely, Wiersinga *et al*. showed that IRAK-1 transcription was downregulated at later times of infection in an experimental mouse model of melioidosis [67]. This depletion of IRAK-1 mRNA agrees with studies in human volunteers injected with LPS [69] and could reflect an early adaptation toward a state of immunotolerance during the course of melioidosis to dampen the overwhelming proinflammatory response. Furthermore, *B. pseudomallei* has mechanisms to evade the host response, e.g., relatively less reactogenic LPS compared to other gram negative pathogens, less stimulatory for TLR4 and lower acute cytokine responses [61,62,67]. COPS1 and AP3B2 are constituents of protein complexes involved in assembling membrane proteins into transport vesicles for sorting to organelles [70–72]. AP3B2 is a subunit of the adaptor protein complex 3 (AP-3), which is required for sorting transmembrane proteins targeted to lysosomes and related organelles. The down regulation in expression of these proteins might disrupt the ability of a host cell to inactivate pathogens, such as *B. pseudomallei*, capable of intracellular survival and persistence [70,71,73].

In addition to being a major stimulator of apoptosis, TNF-α together with IL-1β are proinflammatory cytokines expressed by PBMCs obtained from normal or *B. pseudomallei* -infected humans stimulated by infection or LPS, or in cells from *B. pseudomallei* infected mice [60,62,63,67,68,74]. Moreover, TNF-α, together with IL-1α and IL-18, are enhanced in expression during caspase-1 dependent macrophage death (pyroptosis) induced by *B. pseudomallei* [64].

Thus the macrophage infection model appears to be capable of reproducing aspects of both early and late stages in *B. pseudomallei* infection, *i.e*., the early pro-inflammatory, host-protective immune responses and terminal indicators of immune suppression and cell death. In future studies we hope to determine how bacterial proteins produced inside macrophages, including BPSS1996, influence macrophage function and overall proteome content. The findings of the current study suggest that, in addition to basic questions of pathogenesis, the macrophage cell culture model of *B. pseudomallei* infection may be a useful supplemental tool to evaluate potential efficacy of antimicrobial countermeasures.

## Supplementary Material

Supplemental Material
