# Supplementary material for: A DUF4148 family protein produced inside RAW264.7 cells is a critical Burkholderia pseudomallei virulence factor
Source: Virulence. 2020 Aug 23;11(1):1041–58. doi: 10.1080/21505594.2020.1806675 (PMC7549894; doi:10.1080/21505594.2020.1806675)
Supplement: Supplemental Material [file KVIR_A_1806675_SM0192.zip › Supplemental Figures.PPT]

## Slide 1
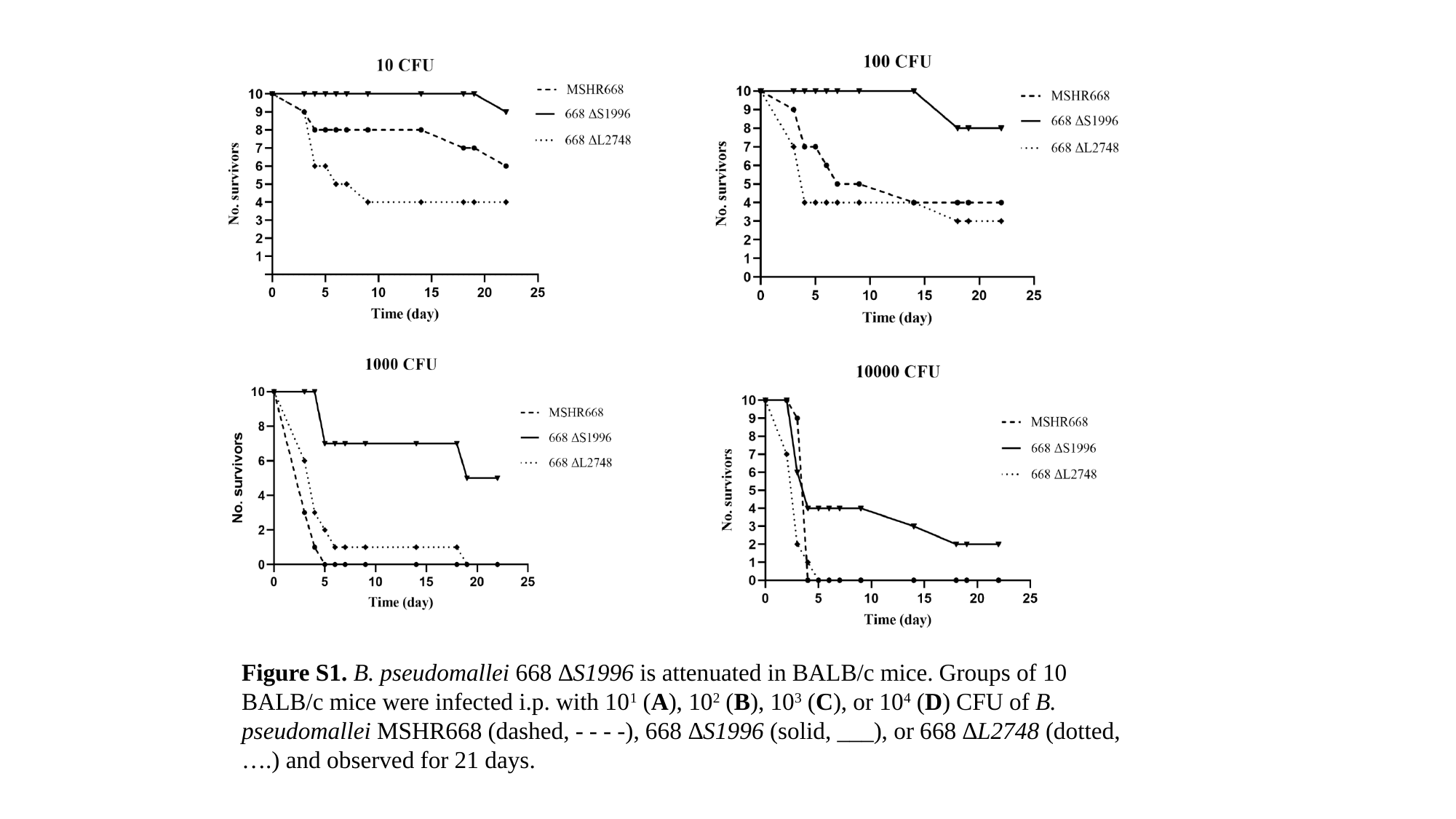

Figure S1. B. pseudomallei 668 ∆S1996 is attenuated in BALB/c mice. Groups of 10 BALB/c mice were infected i.p. with 101 (A), 102 (B), 103 (C), or 104 (D) CFU of B. pseudomallei MSHR668 (dashed, - - - -), 668 ∆S1996 (solid, ­­___), or 668 ∆L2748 (dotted, ….) and observed for 21 days.

## Slide 2
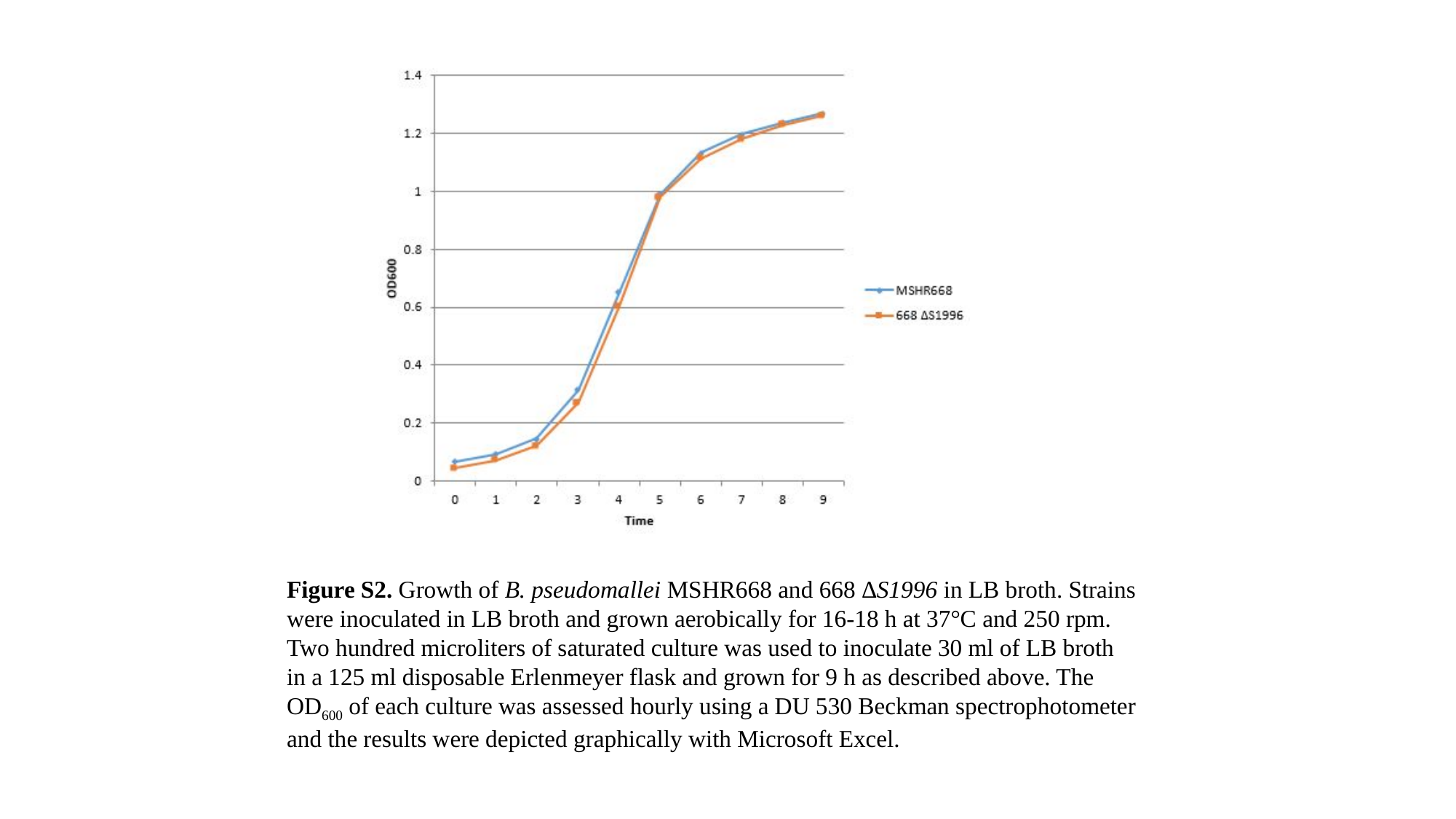

Figure S2. Growth of B. pseudomallei MSHR668 and 668 ∆S1996 in LB broth. Strains were inoculated in LB broth and grown aerobically for 16-18 h at 37°C and 250 rpm. Two hundred microliters of saturated culture was used to inoculate 30 ml of LB broth in a 125 ml disposable Erlenmeyer flask and grown for 9 h as described above. The OD600 of each culture was assessed hourly using a DU 530 Beckman spectrophotometer and the results were depicted graphically with Microsoft Excel.

## Slide 3
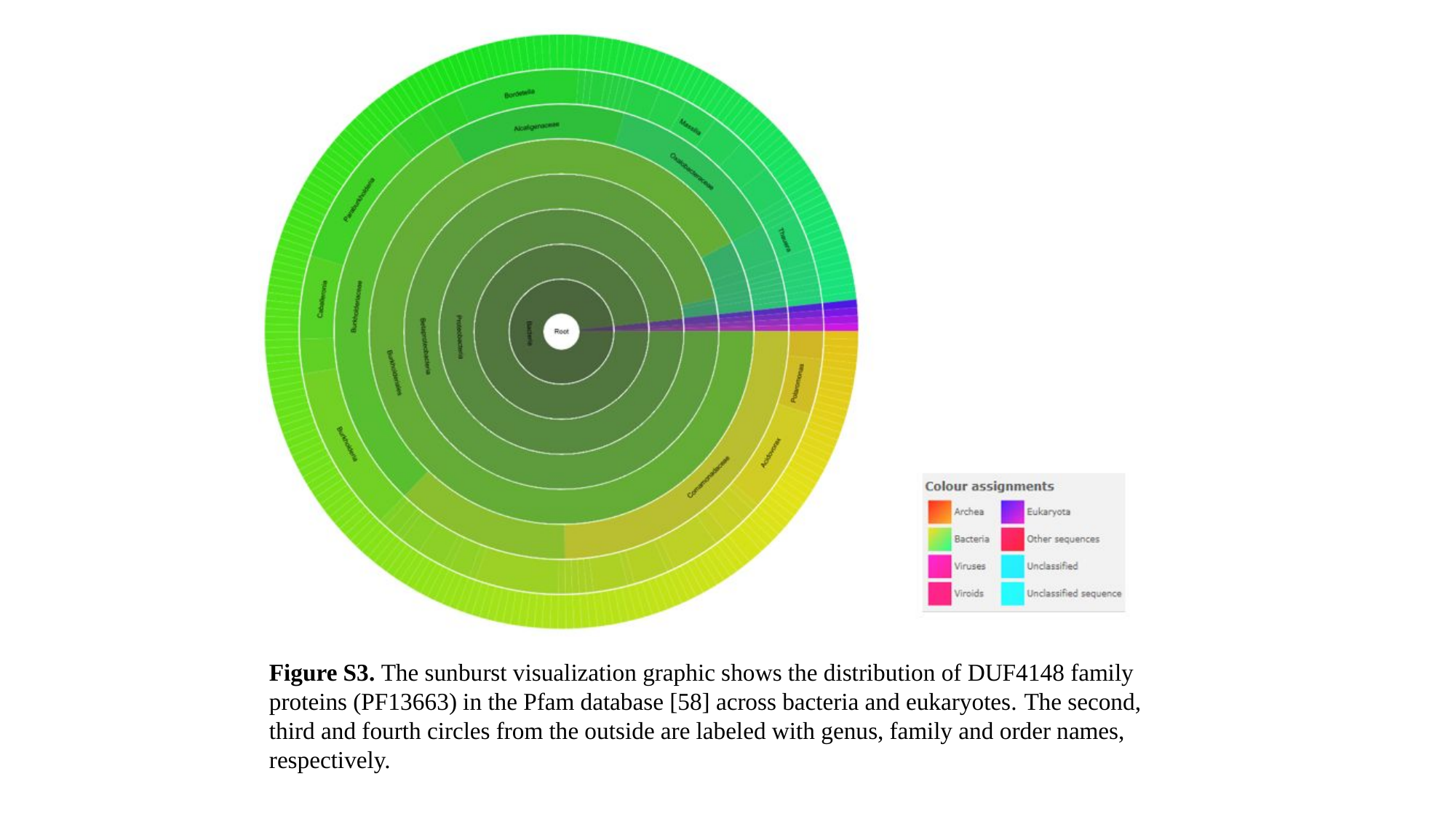

Figure S3. The sunburst visualization graphic shows the distribution of DUF4148 family proteins (PF13663) in the Pfam database [58] across bacteria and eukaryotes. The second, third and fourth circles from the outside are labeled with genus, family and order names, respectively.

## Slide 4
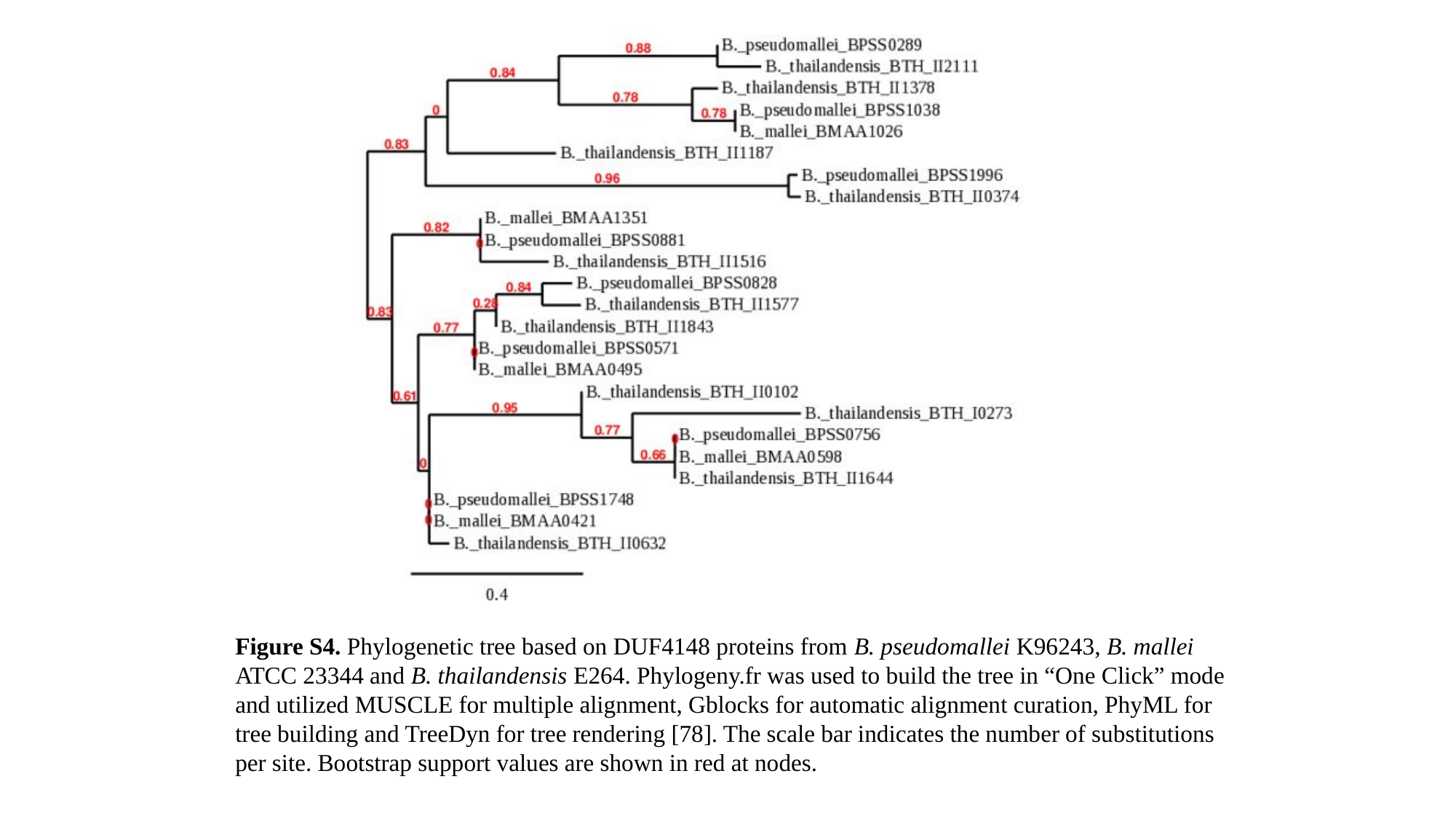

Figure S4. Phylogenetic tree based on DUF4148 proteins from B. pseudomallei K96243, B. mallei ATCC 23344 and B. thailandensis E264. Phylogeny.fr was used to build the tree in “One Click” mode and utilized MUSCLE for multiple alignment, Gblocks for automatic alignment curation, PhyML for tree building and TreeDyn for tree rendering [78]. The scale bar indicates the number of substitutions per site. Bootstrap support values are shown in red at nodes.

## Slide 5
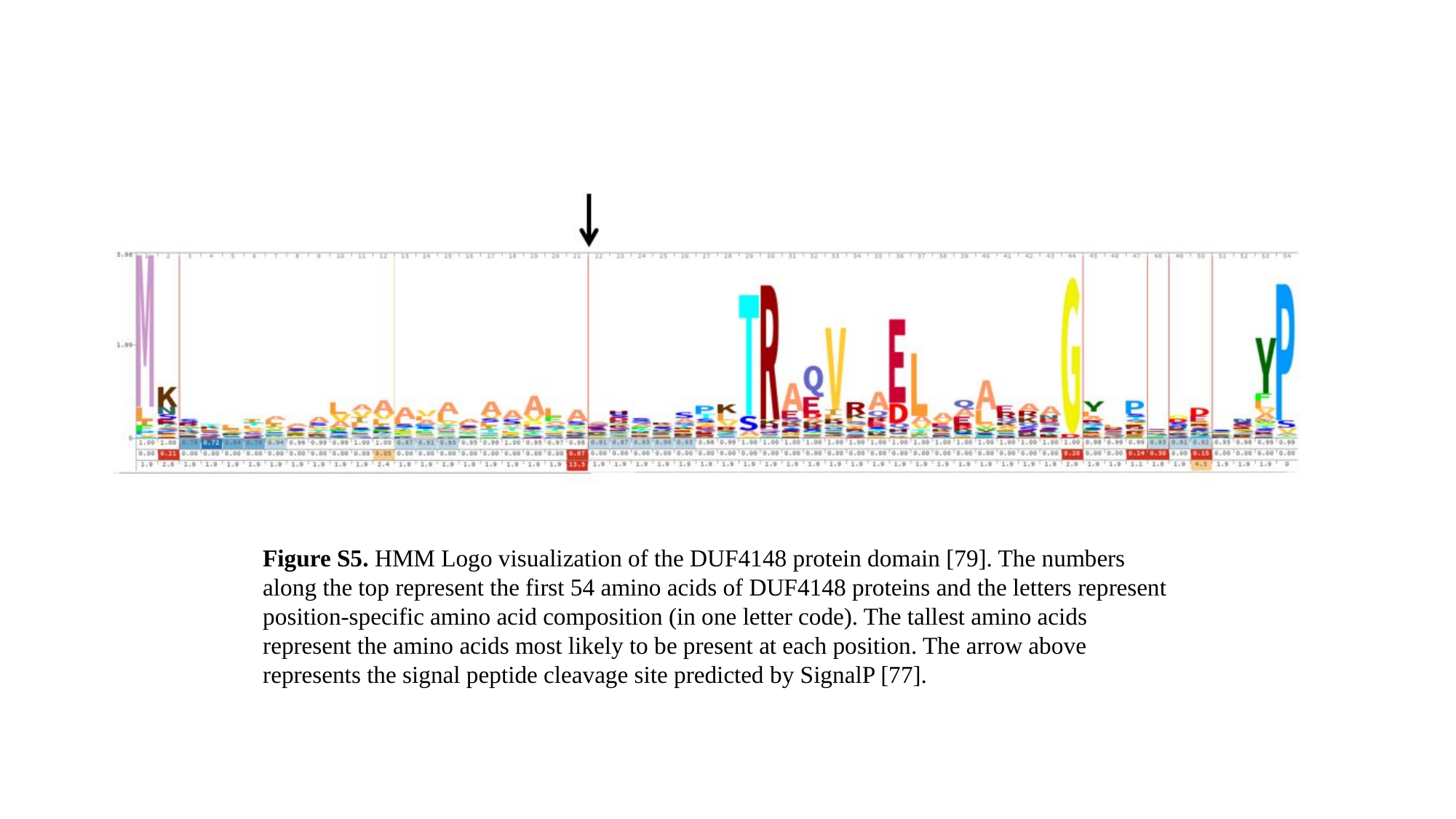

Figure S5. HMM Logo visualization of the DUF4148 protein domain [79]. The numbers along the top represent the first 54 amino acids of DUF4148 proteins and the letters represent position-specific amino acid composition (in one letter code). The tallest amino acids represent the amino acids most likely to be present at each position. The arrow above represents the signal peptide cleavage site predicted by SignalP [77].
