# Supplementary material for: A DUF4148 family protein produced inside RAW264.7 cells is a critical Burkholderia pseudomallei virulence factor
Source: Virulence. 2020 Aug 23;11(1):1041–58. doi: 10.1080/21505594.2020.1806675 (PMC7549894; doi:10.1080/21505594.2020.1806675)
Supplement: Supplemental Material [file KVIR_A_1806675_SM0192.zip › Table S2.docx]

**Table S2**. Proteins exclusively present or absent in *B. pseudomallei*-infected RAW264.7 cells at 12 h post-infection.

| **Protein** | **ID** | **Gene** | **Fold-up in infected** | **Fold-down in infected** |
| --- | --- | --- | --- | --- |
| Protein kinase C binding protein 1 | A2A484 | Prkcbp1 |  | OFF |
| Cell division cycle protein 27 homolog (Part of the anaphase promoting complex or cyclosome (APC/C), a multicomponent ubiquitin ligase and a dominant regulator of the cell cycle. A growing number of viruses have been shown to target the APC/C to create an environment supportive of viral replication.) | A2A6Q5 | Cdc27 |  | OFF |
| Arginine-glutamic acid dipeptide repeats protein (Plays a role as a transcriptional repressor during development. May play a role in the control of cell survival.) | Q80TZ9 | Atr2 (Rere) |  | OFF |
| Transmembrane protein 201 (Isoform Samp1 may define a distinct membrane domain in the vicinity of the mitotic spindle.) | A2A8U2 | Tmem201 (D4Ertd429e) |  | OFF |
| Msx2-interacting protein (May serve as a nuclear matrix platform that organizes and integrates transcriptional responses.) | Q62504 | Spen (Kiaa0929) |  | OFF |
| Stromal antigen 2 (STAG/SA proteins are specific cohesin complex subunits that maintain sister chromatid cohesion in mitosis and meiosis.) | A2AFF6 | Stag2 (RP23-295D9.2-002) |  | OFF |
| E3 ubiquitin-protein ligase BRE1A | Q5DTM8 | Bre1a |  | OFF |
| Caspase recruitment domain family, member 9 | A2AIV8 | Card9 |  | OFF |
| Patatin-like phospholipase domain-containing protein 7 | A2AJ88 | Pnpla7 |  | OFF |
| MCG114640 | G5E920 | Hmg2a |  | OFF |
| Targeting protein for Xklp2 | A2APB8 | Tpx2 |  | OFF |
| RNA polymerase-associated protein RTF1 homolog | A2AQ19 | RP23-22A15.9-001 |  | OFF |
| UPF2 regulator of nonsense transcripts homolog | A2AT37 | RP23-307E15.7-001 |  | OFF |
| Tetratricopeptide repeat protein 4 | Q8R3H9 | Ttc4 |  | OFF |
| Activity-dependent neuroprotective protein | A2BDX0 | Adnp |  | OFF |
| Uncharacterized protein | E9QAQ7 | Arid1a |  | OFF |
| Chromodomain helicase DNA binding protein 6 | A3KFM7 | Chd6 |  | OFF |
| DNA repair protein RAD50 | P70388 | Rad50 |  | OFF |
| Uncharacterized protein | Q3UH70 | Brd4 |  | OFF |
| Mediator of RNA polymerase II transcription subunit 1 | Q925J9 | Crsp210 |  | OFF |
| DNA ligase 3 | P97386 | Lig3 |  | OFF |
| Interleukin-1 receptor-associated kinase 1 | B1AUW6 | Irak1 |  | OFF |
| THO complex subunit 2 | B1AZI6 | Thoc2 |  | OFF |
| PHD finger protein 3 | B2RQG2 | Phf3 |  | OFF |
| RNA polymerase I-specific transcription initiation factor RRN3 | B2RS91 | Rrn3 |  | OFF |
| Nuclear receptor coactivator 5 | Q91W39 | Ncoa5 |  | OFF |
| Tcf20 protein | B9EHJ7 | Tcf20 |  | OFF |
| Uncharacterized protein | B9EIF6 | Zfp36l2 |  | OFF |
| Myc-induced nuclear antigen | Q8CD15 | Mina |  | OFF |
| Uncharacterized protein | D3YU56 | Lemd3 |  | OFF |
| Proteasome subunit beta type | D3YUM8 | Gm4950 |  | OFF |
| Uncharacterized protein | D3YUV9 | Eif4e2 |  | OFF |
| Pogo transposable element with ZNF domain | Q8BZH4 | Kiaa0461 |  | OFF |
| Ubiquitin specific peptidase 16 | G5E860 | Usp16 |  | OFF |
| Uncharacterized protein | D3YXK1 | Samd1 |  | OFF |
| Uncharacterized protein | F6VLR1 | Fam111a |  | OFF |
| Uncharacterized protein | F8VQD1 | Pbrm1 |  | OFF |
| Origin recognition complex subunit 5 | Q9WUV0 | Orc5 |  | OFF |
| Uncharacterized protein | D3Z0X5 | Phldb1 |  | OFF |
| Islet cell autoantigen 1 | P97411 | Ica1 |  | OFF |
| Uncharacterized protein | D3Z357 | Whsc1l1 |  | OFF |
| MCG1219 | D3Z351 | mCG_9102 |  | OFF |
| Zinc finger and BTB domain-containing protein 7A | O88939 | Lrf |  | OFF |
| Putative myosin light chain kinase 3 | Q3UIZ8 | Mylk3 |  | OFF |
| Small ubiquitin-related modifier 2 | P61957 | Smt3b |  | OFF |
| Formin-like protein 3 | Q6ZPF4 | Fmnl3 |  | OFF |
| RIKEN cDNA | Q8BL95 | 1110004E09Rik |  | OFF |
| IQ motif and SEC7 domain-containing protein 1 | E9PUA3 | Iqsec1 |  | OFF |
| Uncharacterized protein | E9PUE3 | Ece2 |  | OFF |
| Uncharacterized protein | E9PWZ7 | Wdr75 |  | OFF |
| Uncharacterized protein | E9PYG5 | Parvg |  | OFF |
| RNA-binding protein 26 | Q6NZN0 | Rbm26 |  | OFF |
| Tetratricopeptide repeat protein 5 | Q99LG4 | Strap |  | OFF |
| Nuclear pore complex protein Nup107 | Q8BH74 | Nup107 |  | OFF |
| Myocyte enhancer factor 2D | Q921S6 | Mef2d |  | OFF |
| Hyaluronan-mediated motility receptor | E9Q837 | Hmmr |  | OFF |
| Protein lin-54 homolog | Q571G4 | Kiaa2037 |  | OFF |
| RNA-binding protein 27 | Q5SFM8 | Kiaa1311 |  | OFF |
| PHD finger protein 10 | Q9D8M7 | Baf45a |  | OFF |
| General transcription factor 3C polypeptide 1 | Q8K284 | Gtf3c1 |  | OFF |
| E3 ubiquitin-protein ligase TRIM33 | Q99PP7 | Kiaa1113 |  | OFF |
| Cyclin-dependent kinase 2-associated protein 1 | O35207 | Cdk2ap1 |  | OFF |
| RNA-binding protein 40 | Q3UZ01 | Kiaa1839 |  | OFF |
| Vesicle transport protein SEC20 | Q6QD59 | Bnip1 |  | OFF |
| Beta-adrenergic receptor kinase 2 | F8VPM8 | Adrbk2 |  | OFF |
| S1 RNA-binding domain-containing protein 1 | Q497V5 | Srbd1 |  | OFF |
| Ras-related protein Rab-3C | P62823 | Rab3c |  | OFF |
| DEAH (Asp-Glu-Ala-His) box polypeptide 36, isoform CRA_a | G3X8Y4 | Ddx36 |  | OFF |
| Apoptotic protease-activating factor 1 (Apaf-1) | O88879 | Apaf1 |  | OFF |
| Helicase (DNA) B | G5E835 | Helb |  | OFF |
| Mitochondrial translation optimization 1 homolog | G5E889 | Mto1 |  | OFF |
| Caspase-6 | O08738 | Casp6 |  | OFF |
| Ribosomal RNA small subunit methyltransferase NEP1 | O35130 | C2f |  | OFF |
| Tumor necrosis factor receptor superfamily member 11A | O35305 | Rank/ Tnfrsf11a |  | OFF |
| Interferon-activable protein 203 | O35368 | Ifi203 |  | OFF |
| Aurora kinase B | O70126 | Ark2 |  | OFF |
| Phosphoglycerate mutase 2 | O70250 | Pgam2 |  | OFF |
| DNA (cytosine-5)-methyltransferase 3A | O88508 | Dnmt3a |  | OFF |
| Bromodomain-containing protein 7 | O88665 | Bp75 |  | OFF |
| Cyclin-K | Q3U3M5 | Ccnk |  | OFF |
| Thymidine kinase, cytosolic | P04184 | Tk1 |  | OFF |
| Interferon-activable protein 204 | P15092 | Ifi204 |  | OFF |
| Non-histone chromosomal protein HMG-14 | P18608 | Hmg14 |  | OFF |
| Alpha-1,3-mannosyl-glycoprotein 2-beta-N-acetylglucosaminyltransferase | P27808 | Gnt1 |  | OFF |
| GTPase KRas | P32883 | Kras |  | OFF |
| Cyclin-A2 | P51943 | Ccna |  | OFF |
| Ubiquitin-conjugating enzyme E2 E3 | P52483 | Ubce4 |  | OFF |
| CCAAT/enhancer-binding protein zeta | P53569 | Cbf2 |  | OFF |
| Tropomyosin beta chain | P58774 | Tpm2 |  | OFF |
| Mortality factor 4-like protein 1 | P60762 | Morf4l1 |  | OFF |
| 40S ribosomal protein S30 | P62862 | Fau |  | OFF |
| Epidermal growth factor receptor kinase substrate 8 | Q08509 | Eps8 |  | OFF |
| RNA pseudouridylate synthase domain-containing protein 2 | Q149F1 | Rpusd2 |  | OFF |
| Cyclin-dependent kinase 12 | Q14AX6 | Cdk12 |  | OFF |
| Cell division cycle-associated protein 2 | Q14B71 | Cdca2 |  | OFF |
| UPF0469 protein KIAA0907 | Q3TCX3 | Kiaa0907 |  | OFF |
| Xylulose kinase | Q3TNA1 | Xylb |  | OFF |
| FAD-dependent oxidoreductase domain-containing protein 1 | Q3TQB2 | Foxred1 |  | OFF |
| Activating transcription factor 7, isoform CRA_a | Q3TZR9 | Atf7 |  | OFF |
| E3 ubiquitin-protein ligase BRE1B | Q3U319 | Bre1b |  | OFF |
| Transforming growth factor beta regulator 1 | Q3UB74 | Niam |  | OFF |
| Polymerase delta-interacting protein 3 | Q8BG81 | Poldip3 |  | OFF |
| Leucine-rich repeat-containing protein 58 | Q3UGP9 | Lrrc58 |  | OFF |
| Ras-responsive element-binding protein 1 | Q3UH06 | Rreb1 |  | OFF |
| Smith-Magenis syndrome chromosomal region candidate gene 8 protein homolog | Q3UMB5 | Smcr8 |  | OFF |
| WASH complex subunit 7 | Q3UMB9 | Kiaa1033 |  | OFF |
| Protein phosphatase 1 regulatory subunit 12C | Q3UMT1 | Mbs85 |  | OFF |
| MKIAA4014 protein | Q571M4 | Akr1c13 |  | OFF |
| Zinc finger FYVE domain-containing protein 26 | Q5DU37 | Kiaa0321 |  | OFF |
| Exocyst complex component 1 | Q8R3S6 | Exoc1 |  | OFF |
| Methyltransferase 10 domain containing protein | Q5SW15 | Mett10d |  | OFF |
| DNA repair protein XRCC1 | Q60596 | Xrcc1 |  | OFF |
| Lymphocyte-specific helicase | Q60848 | Hells |  | OFF |
| SWI/SNF-related matrix-associated actin-dependent regulator of chromatin subfamily D member 1 | Q61466 | Baf60a |  | OFF |
| ATP-dependent RNA helicase DDX3Y | Q62095 | D1Pas1-rs1 |  | OFF |
| Actin-related protein 3B | Q641P0 | Actr3b |  | OFF |
| Zinc finger protein 512 | Q69Z99 | Kiaa1805 |  | OFF |
| Cyclin-dependent kinase 13 | Q69ZA1 | Cdc2l5 |  | OFF |
| Nipped-B-like protein | Q6KCD5 | Nipbl |  | OFF |
| Zinc finger protein 280C | Q6P3Y5 | Kiaa1584 |  | OFF |
| Integrator complex subunit 1 | Q6P4S8 | Ints1 |  | OFF |
| Telomere-associated protein RIF1 | Q6PR54 | Rif1 |  | OFF |
| DNA topoisomerase 2-binding protein 1 | Q6ZQF0 | Kiaa0259 |  | OFF |
| DNA-directed RNA polymerase I subunit RPA34 | Q76KJ5 | Ase1 |  | OFF |
| Mitochondrial carrier homolog 1 | Q791T5 | Mtch1 |  | OFF |
| Importin-8 | Q7TMY7 | Ipo8 |  | OFF |
| Stromal membrane-associated protein 2 | Q7TN29 | Smap1l |  | OFF |
| Histone lysine demethylase PHF8 | Q80TJ7 | Kiaa1111 |  | OFF |
| MKIAA0038 protein | Q80U88 | Eif4h |  | OFF |
| Integrator complex subunit 2 | Q80UK8 | Ints2 |  | OFF |
| UPF0688 protein | Q80WR5 | ? |  | OFF |
| Probable ATP-dependent RNA helicase DDX10 | Q80Y44 | Ddx10 |  | OFF |
| Protein RMD5 homolog A | Q80YQ8 | Rmnd5a |  | OFF |
| Scaffold attachment factor B2 | Q80YR5 | Safb2 |  | OFF |
| Ribonuclease H2 subunit B | Q80ZV0 | Dleu8 |  | OFF |
| Protein MAK16 homolog | Q8BGS0 | Mak16 |  | OFF |
| ADP-ribosylation factor-like protein 6-interacting protein 6 | Q8BH07 | Arl6ip6 |  | OFF |
| Nucleolar complex protein 4 homolog | Q8BHY2 | Noc4l |  | OFF |
| CDKN2A-interacting protein | Q8BI72 | Carf |  | OFF |
| Rho GTPase-activating protein 22 | Q8BL80 | Arhgap22 |  | OFF |
| MCG16669, isoform CRA_f | Q8BLD4 | mCG_16669 |  | OFF |
| General transcription factor 3C polypeptide 4 | Q8BMQ2 | Gtf3c4 |  | OFF |
| Protein FRA10AC1 homolog | Q8BP78 | Fra10ac1 |  | OFF |
| Protein aurora borealis | Q8BS90 | Bora |  | OFF |
| Ribosomal protein S6 kinase beta-1 | Q8BSK8 | Rps6kb1 |  | OFF |
| Leucine-rich repeat and WD repeat-containing protein 1 | Q8BUI3 | Lrwd1 |  | OFF |
| Probable histone-lysine N-methyltransferase NSD2 | Q8BVE8 | Kiaa1090 |  | OFF |
| Retinoblastoma-binding protein 5 | Q8BX09 | Rbbp5 |  | OFF |
| COMM domain-containing protein 2 | Q8BXC6 | Commd2 |  | OFF |
| NF-kappa-B-repressing factor | Q8BY02 | Nkrf |  | OFF |
| Leucine carboxyl methyltransferase 2 | Q8BYR1 | Kiaa0547 |  | OFF |
| Anaphase-promoting complex subunit 2 | Q8BZQ7 | Anapc2 |  | OFF |
| Uncharacterized protein C17orf85 homolog | Q8BZR9 | ? |  | OFF |
| Splicing regulatory glutamine/lysine-rich protein 1 | Q8BZX4 | Sfrs12 |  | OFF |
| Serine/threonine-protein kinase tousled-like 1 | Q8C0V0 | Tlk1 |  | OFF |
| tRNA-splicing endonuclease subunit Sen54 | Q8C2A2 | Sen54 |  | OFF |
| Cleavage stimulation factor subunit 2 tau variant | Q8C7E9 | Cstf2t |  | OFF |
| Transcription elongation factor B polypeptide 3 | Q8CB77 | Tceb3 |  | OFF |
| Pseudouridylate synthase 7 homolog-like protein | Q8CE46 | Pus7l |  | OFF |
| Riboflavin kinase | Q8CFV9 | Rfk |  | OFF |
| RNA-binding protein 28 | Q8CGC6 | Rbm28 |  | OFF |
| Integrator complex subunit 5 | Q8CHT3 | Ints5 |  | OFF |
| BAG family molecular chaperone regulator 4 | Q8CI61 | Bag4 |  | OFF |
| Nucleoside diphosphate-linked moiety X motif 13 | Q8JZU0 | Nudt13 |  | OFF |
| Leucine-rich repeat-containing protein 41 | Q8K1C9 | Lrrc41 |  | OFF |
| DNA-directed RNA polymerase I subunit RPA49 | Q8K202 | Paf53 |  | OFF |
| Probable ATP-dependent RNA helicase DDX52 | Q8K301 | Ddx52 |  | OFF |
| Kinetochore-associated protein NSL1 homolog | Q8K305 | Nsl1 |  | OFF |
| Putative WDC146 | Q8K4P0 | wdc146 |  | OFF |
| HAUS augmin-like complex subunit 3 | Q8QZX2 | Haus3 |  | OFF |
| Splicing factor 3B subunit 4 | Q8QZY9 | Sf3b4 |  | OFF |
| UDP-glucose 4-epimerase | Q8R059 | Gale |  | OFF |
| Integrin-linked kinase-associated serine/threonine phosphatase 2C | Q8R0F6 | Ilkap |  | OFF |
| Nuclear pore complex protein Nup133 | Q8R0G9 | Nup133 |  | OFF |
| Epiplakin | Q8R0W0 | Eppk1 |  | OFF |
| Leydig cell tumor 10 kDa protein homolog | Q8R1F0 | D8Ertd738e |  | OFF |
| Probable RNA-binding protein 19 | Q8R3C6 | Rbm19 |  | OFF |
| Nucleolar protein 6 | Q8R5K4 | Nol6 |  | OFF |
| U3 small nucleolar RNA-associated protein 6 homolog | Q8VCY6 | Mhat |  | OFF |
| Myotubularin-related protein 6 | Q8VE11 | Mtmr6 |  | OFF |
| Ankyrin repeat domain-containing protein 49 | Q8VE42 | Ankrd49 |  | OFF |
| U3 small nucleolar ribonucleoprotein protein IMP4 | Q8VHZ7 | D1Wsu40e |  | OFF |
| Nucleolar complex protein 3 homolog | Q8VI84 | Ad24 |  | OFF |
| Putative hexokinase HKDC1 | Q91W97 | Hkdc1 |  | OFF |
| Thymocyte nuclear protein 1 | Q91YJ3 | Thy28 |  | OFF |
| Suppressor of SWI4 1 homolog | Q91YU8 | Ppan |  | OFF |
| COP9 signalosome complex subunit 1 | Q99LD4 | Cops1 |  | OFF |
| Polyribonucleotide 5-hydroxyl-kinase Clp1 | Q99LI9 | Clp1 |  | OFF |
| DNA replication complex GINS protein SLD5 | Q99LZ3 | Gins4 |  | OFF |
| Kinetochore protein Nuf2 | Q99P69 | Cdca1 |  | OFF |
| Maged2 protein | Q9ER67 | Maged2 |  | OFF |
| E3 ubiquitin-protein ligase RING2 | Q9CQJ4 | DinG |  | OFF |
| Zinc finger HIT domain-containing protein 3 | Q9CQK1 | Trip3 |  | OFF |
| Ribosome biogenesis protein NSA2 homolog | Q9CR47 | Nsa2 |  | OFF |
| PRKR-interacting protein 1 | Q9CWV6 | Prkrip1 |  | OFF |
| Ribosome biogenesis regulatory protein homolog | Q9CYH6 | MNCb-2643 |  | OFF |
| Uncharacterized protein C3orf26 homolog | Q9CZT6 | ? |  | OFF |
| Cell division cycle-associated protein 7 | Q9D0M2 | Cdca7 |  | OFF |
| 60S ribosomal protein L7-like 1 | Q9D8M4 | Rpl7l1 |  | OFF |
| Peroxisomal carnitine O-octanoyltransferase | Q9DC50 | Cot |  | OFF |
| Protein phosphatase inhibitor 2 | Q9DCL8 | Ppp1r2 |  | OFF |
| Sentrin-specific protease 3 | Q9EP97 | Senp3 |  | OFF |
| ETS-related transcription factor Elf-2 | Q9JHC9 | Elf2 |  | OFF |
| Lysine-specific demethylase NO66 | Q9JJF3 | MNCb-7109 |  | OFF |
| DNA mismatch repair protein Mlh1 | Q9JK91 | Mlh1 |  | OFF |
| AP-3 complex subunit beta-2 (AP-3 appears to be involved in the sorting of a subset of transmembrane proteins targeted to lysosomes and lysosome-related organelles.) | Q9JME5 | Ap3b2 |  | OFF |
| Inner centromere protein | Q9WU62 | Incenp |  | OFF |
| DNA polymerase epsilon catalytic subunit A | Q9WVF7 | Pole |  | OFF |
| Rac GTPase-activating protein 1 | Q9WVM1 | Mgcracgap |  | OFF |
| WD repeat-containing protein 46 | Q9Z0H1 | Bing4 |  | OFF |
| CAP-Gly domain-containing linker protein 2 | Q9Z0H8 | Clip2 |  | OFF |
| Xenotropic and polytropic retrovirus receptor 1 | Q9Z0U0 | Syg1 |  | OFF |
| Myotubularin-related protein 1 (Lipid phosphatase that acts on phosphatidylinositol 3-phosphate and phosphatidylinositol (3,5)-bisphosphate.) | Q9Z2C4 | Mtmr1 |  | OFF |
| Methyl-CpG-binding protein 2 | Q9Z2D6 | Mecp2 |  | OFF |
| DNA topoisomerase 3-beta-1 | Q9Z321 | Top3b |  | OFF |
| Inositol 1,4,5-trisphosphate receptor type 2 | Q9Z329 | Itpr2 |  | OFF |
| Heat shock protein 90kDa alpha (Cytosolic), class A member 1 | A2A6A2 | Hsp90aa1 | ON |  |
| Chromodomain-helicase-DNA-binding protein 7 | A2AJK6 | Chd7 | ON |  |
| ARF6 guanine nucleotide exchange factor IQArfGEF | E9QAD8 | IQArfGEF | ON |  |
| Guanylate binding protein 1 | A4UUI2 | Gbp1 | ON |  |
| Uncharacterized protein (vesicle-mediated transport) | D3YTU0 | Vamp1 | ON |  |
| Microtubule-associated protein RP/EB family member 3 | Q6PER3 | Mapre3 | ON |  |
| Rho guanine nucleotide exchange factor 7 | D3Z0V2 | Arhgef7 | ON |  |
| Uncharacterized protein (microtubule-based movement) | D3Z2X2 | Dnhd1 | ON |  |
| Uncharacterized protein | Q8BG33 | Hnt | ON |  |
| Serine/threonine-protein kinase WNK2 | E9QM73 | Wnk2 | ON |  |
| Protein Fry | E9Q8I9 | Fry | ON |  |
| H^+^/Cl^-^ exchange transporter 7 (contributes to the acidification of the lysosome lumen) | O70496 | Clc7 | ON |  |
| Protein Kif14 (microtubule-based movement) | E9Q3T3 | Kif14 | ON |  |
| Probable phospholipid-transporting ATPase IA (phospolipid 'flippase' activity, driven by the hydrolysis of ATP) | Q8BR88 | Atp8a1 | ON |  |
| Protein piccolo | E9QK94 | Pclo | ON |  |
| Dynamin-1 (involved in receptor-mediated endocytosis) | P39053 | Dnm | ON |  |
| Uncharacterized protein (putative serine/threonine-protein kinase) | F6V672 | Mapk11 | ON |  |
| Heterogeneous nuclear ribonucleoprotein M | F6W322 | Hnrnpm | ON |  |
| Mast cell surface glycoprotein Gp49A (a 49-kDa type I transmembrane glycoprotein, a member of the Ig-like receptors) | F8WJB7 | Gp49a | ON |  |
| Inter-alpha trypsin inhibitor, heavy chain 2 (plasma serine protease inhibitor, overexpression in U251 glioma cells promoted cell-cell adhesion) | G3X977 | Itih2 | ON |  |
| Syntaxin-1A (exocytosis function) | O35526 | Stx1a | ON |  |
| Complement C3 | P01027 | C3 | ON |  |
| Interleukin-1 alpha | P01582 | Il1a | ON |  |
| Major prion protein | P04925 | Prnp | ON |  |
| Granulocyte colony-stimulating factor (G-CSF) | P09920 | Csf3 | ON |  |
| Interleukin-1 beta | P10749 | Il1b | ON |  |
| C-X-C motif chemokine 2 (Chemotactic for human polymorphonuclear leukocytes) | P10889 | Cxcl2 | ON |  |
| Plasminogen activator inhibitor 2, macrophage (acutely upregulated in pregnancy, inflammation, infection, and other pathophysiological conditions) | P12388 | Pai2 | ON |  |
| Tumor necrosis factor receptor superfamily member 1B (Receptor with high affinity for TNFSF2/TNF-alpha) | P25119 | Tnfr2 | ON |  |
| Protein S100-A8 (Calcium-binding protein that has antimicrobial activity towards bacteria and fungi. Important for resistance to invasion by pathogenic bacteria. Up-regulates transcription of genes that are under the control of NF-kappa-B.) | P27005 | Caga | ON |  |
| Tumor necrosis factor receptor superfamily member 5 | P27512 | Cd40 | ON |  |
| MKIAA0531 protein (microtubule-based movement) | Q8CHF1 | Kif5c | ON |  |
| Vascular endothelial growth factor receptor 1 (VEGFR-1; Tyrosine-protein kinase that acts as a cell-surface receptor for VEGFA, VEGFB and PGF) | P35969 | Emrk2 | ON |  |
| TNF receptor-associated factor 1 (Adapter molecule that regulates the activation of NF-kappa-B and JNK. Plays a role in the regulation of cell survival and apoptosis.) | P39428 | Traf1 | ON |  |
| Synaptotagmin-2 (role in the membrane interactions during trafficking of synaptic vesicles at the active zone of the synapse) | P46097 | Syt2 | ON |  |
| C-C chemokine receptor type 1 (Receptor for a C-C type chemokine. Binds to MIP-1-alpha, RANTES, and less efficiently, to MIP-1-beta or MCP-1 and subsequently transduces a signal by increasing the intracellular calcium ions level.) | P51675 | Ccr1 | ON |  |
| Lumican (Secreted; Binds to laminin) | P51885 | Lcn | ON |  |
| T-lymphocyte activation antigen CD80 (T-cell proliferation and cytokine production is induced by the binding of CD28 or CTLA-4 to this receptor.) | Q00609 | B7 | ON |  |
| Junction plakoglobin (Cell adhesion) | Q02257 | Jup | ON |  |
| Myelin oligodendrocyte glycoprotein | Q3UY21 | Mog | ON |  |
| Copine-6 (May function in membrane trafficking.) | Q9Z140 | Cpne6 | ON |  |
| Tyrosine-protein phosphatase non-receptor type 18 (Uncharacterized protein) | Q3V441 | Ptpn18 | ON |  |
| Guanylate-binding protein 4 (Binds GTP, GDP and GMP. Hydrolyzes GTP very efficiently; GDP rather than GMP is the major reaction product. Plays a role in erythroid differentiation.) | Q61107 | Gbp4 | ON |  |
| Podoplanin (May be involved in cell migration and/or actin cytoskeleton organization.) | Q62011 | Pdpn (Gp38) | ON |  |
| Sepiapterin reductase (Catalyzes the final one or two reductions in tetra-hydrobiopterin biosynthesis to form 5,6,7,8-tetrahydrobiopterin, which is a cofactor for the production of nitric oxide (NO) by the nitric oxide synthases) | Q64105 | Spr | ON |  |
| Tenascin (Extracellular matrix protein; Ligand for integrins alpha-8/beta-1, alpha-9/beta-1, alpha-V/beta-3 and alpha-V/beta-6.) | Q80YX1 | Tnc | ON |  |
| 3-ketoacyl-CoA thiolase A, peroxisomal (Uncharacterized protein) | Q8BLD7 | Acaa1 | ON |  |
| NADP-dependent malic enzyme, mitochondrial | Q8BMF3 | Me3 | ON |  |
| EGF-containing fibulin-like extracellular matrix protein 1 (May play a role in cell adhesion and migration.) | Q8BPB5 | Efemp1 | ON |  |
| Solute carrier family 15 member 3 (Lysosome membrane protein; Proton oligopeptide cotransporter. Transports free histidine and certain di- and tripeptides) | Q8BPX9 | Slc15a3 | ON |  |
| Radical S-adenosyl methionine domain-containing protein 2 (Involved in antiviral defense. May impair virus budding by disrupting lipid rafts at the plasma membrane, a feature which is essential for the budding process of many viruses.) | Q8CBB9 | Rsad2 | ON |  |
| Tensin-like C1 domain-containing phosphatase (Regulates cell motility and proliferation. May have phosphatase activity.) | Q8CGB6 | Tenc1 | ON |  |
| Protein lin-7 homolog A (Exocytosis) | Q8JZS0 | Lin7a | ON |  |
| Neurocalcin-delta (Binds three calcium ions) | Q91X97 | Ncald | ON |  |
| Secernin-1 (Regulates exocytosis in mast cells. Increases both the extent of secretion and the sensitivity of mast cells to stimulation with calcium) | Q9CZC8 | Scrn1 | ON |  |
| Vitamin K-dependent gamma-carboxylase (Mediates the vitamin K-dependent carboxylation of glutamate residues to calcium-binding gamma-carboxyglutamate (Gla) residues with the concomitant conversion of the reduced hydroquinone form of vitamin K to vitamin K epoxide.) | Q9QYC7 | Ggcx | ON |  |
| Thrombospondin-4 (Adhesive glycoprotein that mediates cell-to-cell and cell-to-matrix interactions. Can bind to fibrinogen, fibronectin, laminin and type V collagen) | Q9Z1T2 | Thbs4 | ON |  |
| SNARE-associated protein Snapin (May have a role in the mechanisms of SNARE-mediated membrane fusion in non-neuronal cells.) | Q9Z266 | Snap25bp | ON |  |
